# Computational Analysis of Electron-Donating and Withdrawing Effects on Asymmetric Viologens for Enhanced Electrochromic Performance

**DOI:** 10.3390/ijms262010137

**Published:** 2025-10-18

**Authors:** Gulzat Nuroldayeva, Mannix P. Balanay

**Affiliations:** 1 Department of Chemistry, Nazarbayev University, 53 Kabanbay Batyr Ave., Astana 010000, Kazakhstan; gulzat.nuroldayeva@nu.edu.kz; 2 National Laboratory of Astana, Nazarbayev University, 53 Kabanbay Batyr Ave., Astana 010000, Kazakhstan

**Keywords:** asymmetric viologen, electrochromic materials, DFT, TD-DFT, electron-withdrawing, electron-donating

## Abstract

Viologens are promising candidates for next-generation electrochromic devices due to their reversible color changes, low operating voltages, and structural tunability. However, their practical performance is often constrained by limited color range, stability issues, and poor charge delocalization. In this study, we present a detailed density functional theory (DFT) and time-dependent DFT (TD-DFT) investigation of asymmetric viologens based on the Benzyl-4,4′-dipyridyl-R (BnV-R) framework. A series of electron-donating and electron-withdrawing substituents (CN, COOH, PO_3_H_2_, CH_3_, OH, NH_2_) were introduced via either benzyl or phenyl linkers. Geometry optimizations for neutral, radical cationic, and dicationic states were performed at the CAM-B3LYP/6-31+G(d,p) level with C-PCM solvent modeling. Electronic structure, frontier orbital distributions, and redox potentials were correlated with substituent type and linkage mode. Natural Bond Orbital analysis showed that electron-withdrawing groups stabilize reduced states, while electron-donating groups enhance intramolecular charge transfer and switching kinetics. TD-DFT calculations revealed significant bathochromic and hyperchromic shifts dependent on substitution patterns, with phenyl linkers promoting extended conjugation and benzyl spacers minimizing aggregation. Radical cation stability, quantified via Δ*E_red_* and comproportionation constants, highlighted cyano- and amine-substituted systems as particularly promising. These insights provide predictive design guidelines for tuning optical contrast, coloration efficiency, and electrochemical durability in advanced electrochromic applications.

## 1. Introduction

Electrochromism is a unique property exhibited by certain materials, characterized by their ability to reversibly change color or optical properties when subjected to an external electric field [1]. This behavior distinguishes electrochromic materials from conventional compounds, as they can undergo controlled and repeatable optical transitions in response to electrical stimulation. Due to this electrically tunable optical response, electrochromic materials have found widespread application across a broad range of technologies. Their utility is particularly notable in energy-efficient systems, where modulation of light and heat transmission can lead to significant energy savings. Common applications include smart windows for buildings and vehicles, self-dimming rear-view mirrors in automobiles, antiglare displays, adaptive sunglasses, and electronic display technologies, among others [2,3,4,5,6,7].

Among the various classes of electrochromic materials, viologen-based systems have garnered significant attention due to their superior electrochemical and optical properties. Viologens, typically derivatives of 4,4′-bipyridinium, stand out due to several advantages, including their fast-switching behavior, pronounced color changes, and, importantly, their ability to function at low operating voltages, enhancing both energy efficiency and device longevity [8]. Despite these benefits, viologen-based electrochromic materials still face several challenges. These include limited color diversity, environmental sensitivity, and long-term stability under operational conditions [9,10].

Recent advancements in the field have focused on overcoming these limitations through molecular engineering strategies. These involve the incorporation of electron-donating or electron-withdrawing substituents, the use of extended π-conjugated systems, and strategic modifications of the viologen core structure [11,12,13]. In particular, asymmetric viologens (AV), which feature two distinct substituents on the bipyridinium core, have emerged as a promising subclass with enhanced electrochromic performance compared to their symmetric counterpart [14,15,16].

Asymmetric viologens offer several advantages derived from their unique structural characteristics. The presence of two different substituents enables precise tuning of the molecule’s electronic properties, allowing for better modulation of redox behavior and optical absorption features [17,18,19]. These derivatives typically possess three distinct and reversible redox states, enabling dynamic and multi-step color modulation, which is critical for high-performance electrochromic devices. Furthermore, the electronic asymmetry introduced by these substituents can enhance intramolecular charge delocalization and broaden the absorption spectrum, thereby enabling richer and more diverse color transitions. Asymmetric viologens also demonstrate improved cycle stability, a key factor for long-term practical use.

In solid-state applications, the intrinsic dipole moments of asymmetric viologens can promote stronger intermolecular interactions and more uniform film formation, which are essential for device performance and durability. Theoretical investigations, particularly those based on density functional theory (DFT), have corroborated these experimental findings. DFT studies indicate that asymmetric substitution can lead to favorable shifts in the energy levels of frontier molecular orbitals, namely the highest occupied molecular orbital (HOMO) and lowest unoccupied molecular orbital (LUMO), as well as optimized absorption wavelengths and electrochemical stability [20].

**Figure 1:**
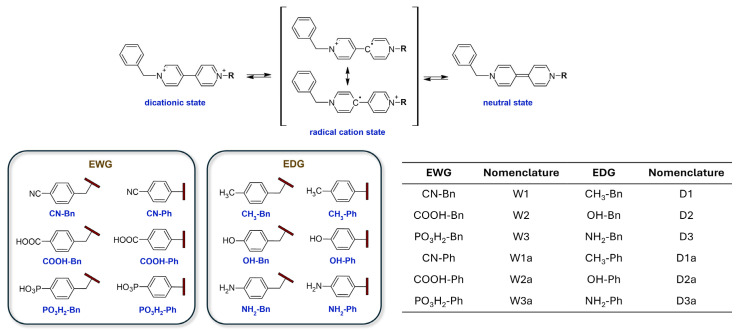
(**Top**) Redox states of the benzyl-4,4′-dipyridyl-substituted compounds. (**Bottom**) Chemical structures of the substituents used in this study, categorized as electron-donating (EDG) or electron-withdrawing groups (EWG), along with their corresponding nomenclature.

In this work, we focus on asymmetric viologen derivatives built around the Benzyl-4,4′-dipyridyl-R (Bn-V-R) framework, where one nitrogen atom of the bipyridyl core is linked to a benzyl group (Bn), and the R substituent varies systematically between electron-withdrawing groups (EWGs) and electron-donating groups (EDGs) (Figure 1). The choice of benzyl as a substituent is particularly advantageous due to the relative ease of its incorporation through straightforward synthetic routes. The benzyl moiety can be introduced via simple nucleophilic substitution reactions, typically involving benzyl halides and the bipyridyl nitrogen, often under mild conditions that promote high yields and operational simplicity. This ease of synthesis allows for efficient generation of a diverse library of asymmetric viologen derivatives without necessitating complex or labor-intensive synthetic steps [21].

Furthermore, the presence of the methylene (-CH_2_-) spacer between the bipyridyl nitrogen and the aromatic ring in the benzyl group introduces unique electronic and steric effects compared to direct phenyl substitution (Ph-V-R analogues). The flexible methylene linker not only reduces conjugation between the nitrogen lone pair and the aromatic system but also modulates the electronic communication across the molecule, potentially influencing the redox behavior and stability of the viologen core. This spacer also adds a degree of conformational freedom, which can affect the steric environment around the redox-active center, influencing intermolecular interactions and solubility profiles.

For comparison, phenyl (Ph)-substituted analogues are also investigated, allowing us to assess the impact of the methylene spacer in benzyl on electronic communication, steric effects, and redox properties. The substituents considered include EWGs (CN-Bn, COOH-Bn, PO_3_H_2_-Bn; CN-Ph, COOH-Ph, PO_3_H_2_-Ph) and EDGs (CH_3_-Bn, OH-Bn, NH_2_-Bn; CH_3_-Ph, OH-Ph, NH_2_-Ph). By systematically varying R groups within these frameworks, we can elucidate how the interplay between electronic effects and structural features influences the overall electrochemical behavior and functional properties of the viologen derivatives.

Electron-withdrawing substituents are known to stabilize reduced viologen states by lowering electron density at the bipyridinium core, thereby enhancing redox tunability and producing more intense color changes upon reduction [22]. In contrast, electron-donating groups increase electron density, resulting in lower oxidation potential and improved stability of oxidized species, often accompanied by less pronounced optical shifts [23,24]. This duality underscores the critical role of substituent identity and position in tuning electrochemical and optical properties for electrochromic applications.

To quantitatively elucidate these effects, we employ DFT at the CAM-B3LYP/6-31+G(d,p) level, incorporating the conductor-like polarizable continuum model (C-PCM) to simulate solvent and environmental influences typical of electrochemical systems. This computational framework enables detailed evaluation of frontier molecular orbitals, HOMO-LUMO gaps, redox potential, solvation energies, and charge redistribution upon oxidation and reduction.

By optimizing the geometries of both neutral and cationic states, this study aims to correlate substituent effects and linker identity with key electrochromic performance parameters, including coloration efficiency, switching speed, and material stability. The comparative analysis of phenyl- versus benzyl-linked viologens further provides insight into how the methylene spacer modulates electronic delocalization, steric hindrance, and charge-transfer dynamics. Collectively, these insights guide the rational design of asymmetric viologens with balanced optical contrast, redox behavior, and synthetic feasibility for next-generation electrochromic devices.

## 2. Results and Discussion

### 2.1. Electronic Structure and Bonding Analysis of Asymmetric Viologen

Strategic design of asymmetric viologens by incorporating a combination of electron-donating (D) and electron-withdrawing (A) substituents on the N-positions enables fine-tuning of redox behavior, charge distribution, and switching kinetics, which are the key parameters for advanced electrochromic device functionality. In this study, a controlled donor–acceptor design was implemented by pairing Bn (an electron-donating spacer) with substituents of varying electronic nature, either directly or through a phenyl linker. Compounds were thus classified according to the electronic nature of their terminal groups: D–V–D systems (e.g., D1–D3 and D1a–D3a) featuring dual donor substitution, and D–V–A systems (e.g., W1-W3 and W1a-W3a) comprising both electron-donating and electron-withdrawing motifs. A–V–A systems were not included in this study, as their symmetric acceptor-rich nature does not align with the objective of probing electronic asymmetry. Moreover, such configurations often exhibit diminished electrochromic performance due to poor charge delocalization, limited optical tunability, and unfavorable redox characteristics [25].

Electron-withdrawing groups such as CN, COOH, and PO_3_H_2_ were selected to lower the LUMO energy level, enhance electron affinity, and facilitate reduction. Acidic substituents (W2, W2a, W3, W3a) further provide anchoring capabilities and proton-donor character, supporting improved film formation and ion conductivity. On the other hand, CH_3_, OH, and NH_2_ groups function as electron donors, elevating HOMO energies and promoting intramolecular charge transfer. Among them, NH_2_-substituted derivatives (D3 and D3a) exhibit the strongest donor behavior due to resonance delocalization and lone pair participation. Additionally, the use of phenyl linkers (in “a”-series compounds) introduces extended π-conjugation, enhancing delocalization and molecular rigidity, while the benzyl-spaced systems increase conformational flexibility and reduce aggregation by disrupting planarity. This matrix of substituent and linker variations allows a systematic exploration of how electronic and steric effects influence electrochemical and optical properties.

The optimized geometrical parameters of asymmetric viologens in their three redox states (neutral, radical cation, and dication) demonstrate distinct and progressive structural transformations that are strongly influenced by both the electronic character and steric bulk of the substituents (Table 1). In the neutral state, the N–C bond lengths (N1–C2 and N1′–C2′) are uniformly short (~1.44–1.45 Å), indicative of effective π-conjugation across the bipyridyl system. The central C5–C5′ bond (~1.37 Å) retains partial double-bond character, and the minimal torsion angles across the C4–C5–C5′–C4′ dihedral (~0.1–1.3°) confirm that the bipyridyl core remains essentially planar. However, torsional angles involving the N-substituents (C1–C2–N1–C3 and C3′–N1′–C2′–C1′) deviate significantly from planarity, reflecting rotational freedom around the N–C bonds.

Most derivatives such as W1–W3 and D1–D2 exhibit torsion angles near 85–87°, indicating a quasi-perpendicular orientation of the N-substituents relative to the planar core. This likely minimizes steric and electronic repulsion with the π-system. In contrast, D3 (NH_2_-Bn) shows a significantly reduced dihedral angle (~60°), suggesting that the strong electron-donating amino group enhances conjugation or intramolecular interactions, favoring a more coplanar alignment.

Phenyl-substituted derivatives display even lower torsional angles (~29–42°), reflecting a greater tendency toward coplanarity. Electron-withdrawing groups (e.g., W1a: CN-Ph, W2a: COOH-Ph, W3a: PO_3_H_2_-Ph) show the smallest angles (~29–30°), while electron-donating groups (e.g., D1a: CH_3_-Ph, D2a: OH-Ph, D3a: NH_2_-Ph) yield slightly larger angles (~37–42°). These trends are consistent with electronic effects: electron-withdrawing substituents stabilize the partial positive charge at nitrogen via inductive and resonance contributions, promoting stronger π-conjugation and enhanced planarity. Conversely, electron-donating groups may introduce lone-pair repulsion or reduce conjugation efficiency, favoring more twisted conformations. Overall, the rigidity of the phenyl ring supports planarity, while substituent electronics modulate the exact torsional profile.

Upon single-electron oxidation to the radical cation state, distinct structural changes emerge due to altered electron distribution. N–C bond lengths increase by ~0.02–0.04 Å, indicating partial bond weakening through charge delocalization. The central C5–C5′ bond elongates to ~1.42–1.43 Å, reflecting reduced double-bond character. More notably, the substituent torsion angles (C1–C2–N1–C3 and C3′–N1′–C2′–C1′) decrease substantially, often falling to 45–56°, signifying partial planarization or increased flexibility. For example, W3 and D3 exhibit angles around 50° and 38°, respectively, while phenyl derivatives (W1a, W2a) maintain intermediate torsions (~45–50°). This shift reflects a balance: oxidation enhances the desire for conjugation, reducing dihedral angles to improve orbital overlap, while steric hindrance continues to limit full coplanarity.

Interestingly, the bipyridyl core remains planar (C4–C5–C5′–C4′ ≈ 0°) across all derivatives, suggesting that conformational reorganization in the radical cation state is localized primarily at the N-substituent interface. Both electron-withdrawing and electron-donating groups exhibit similar reductions in torsion, albeit with subtle variations attributable to differences in conjugation and steric effects.

Further oxidation to the dicationic state induces pronounced geometric reorganization. N–C bond lengths extend to ~1.52–1.54 Å, indicating a significant reduction in bond order and localization of positive charge at the nitrogen centers. The central C5–C5′ bond also lengthens to ~1.487 Å, consistent with near-complete loss of π-bond character and electronic decoupling of the pyridyl units. A key observation of the dicationic state is the dramatic flattening of the substituent torsion angle (C1–C2–N1–C3), which drops to ~1–6° across all derivatives. This near-planarity reflects enhanced π-conjugation between the electron-deficient nitrogen and its substituent, favoring extended delocalization to stabilize the dicationic charge.

In contrast, the bipyridyl core becomes twisted, with C4–C5–C5′–C4′ dihedral angles increasing to ~40–42°. This out-of-plane distortion likely arises from electrostatic repulsion between the two positively charged pyridinium units, which adopt a staggered conformation to minimize Coulombic strain at the expense of π-conjugation through the bridge.

For phenyl-substituted derivatives (W1a–W3a, D1a–D3a), torsion angles at the N-substitution site increase to ~45–57°, indicating a return to moderately twisted geometries. Despite the strong electron deficiency at nitrogen, steric bulk and rigidity of the substituents preclude full coplanarity, resulting in intermediate torsions—slightly higher than those observed in the radical cation state. This suggests a compromise between conjugative stabilization and steric constraints.

Overall, these observations underscore the redox-responsive conformational flexibility of viologen scaffolds. Oxidation induces a systematic evolution from planar, conjugated structures to twisted, charge-separated geometries. The electronic and steric characteristics of the substituents modulate this behavior, offering a tunable platform for designing viologen-based materials with customized electronic, optical, and redox properties.

The second-order perturbation analysis based on Natural Bond Orbital (NBO) calculations provides critical insights into the intramolecular donor–acceptor interactions that govern the stability of asymmetric viologens (Supplementary Information, Tables S1–S12). These interactions are primarily driven by electron delocalization from bonding orbitals (BD) to antibonding orbitals (BD*), which contributes significantly to the thermodynamic stabilization of the molecular systems studied.

For the benzyl-substituted viologens, most compounds exhibit stabilization energies (E^2^) in the range of ~1.7 to 4.1 kcal·mol^−1^. Among these, W2 (COOH–Bn) and W3 (PO_3_H_2_–Bn) show the highest stabilization energies of 4.07 and 3.97 kcal·mol^−1^, respectively. These elevated values reflect the strong electron-withdrawing character of the carboxylic acid and phosphonic acid groups, which enhance delocalization across the viologen framework. This delocalization mitigates charge buildup on the core, thereby improving thermodynamic stability. In contrast, D2 (OH–Bn) and D3 (NH_2_–Bn), which contain electron-donating substituents, exhibit slightly lower stabilization energies (~3.6–3.9 kcal·mol^−1^). This reduction suggests weaker donor–acceptor interactions, likely due to less effective orbital overlap between the substituent and the viologen core, resulting in comparatively reduced delocalization and molecular stabilization.

The phenyl-substituted analogues generally show stronger donor–acceptor interactions, attributed to enhanced π-conjugation between the phenyl ring and the viologen core. This is particularly evident for D2a (OH–Ph) and D3a (NH_2_–Ph), where significant BD → BD* interactions are facilitated through extended conjugation. Notably, D3a displays a remarkably high stabilization energy (~17.4 kcal·mol^−1^), indicating extensive delocalization. However, such pronounced delocalization may also induce charge localization or redistribution issues, potentially compromising redox and structural stability during cycling. Conversely, phenyl derivatives with electron-withdrawing groups, such as W2a (COOH–Ph) and W3a (PO_3_H_2_–Ph), exhibit more moderate stabilization energies (~3.8–3.9 kcal·mol^−1^). These values suggest a balanced electronic environment where delocalization is sufficient to stabilize the viologen core, without leading to over-delocalization that might destabilize the redox-active framework.

Thus, the NBO analysis identifies W2 (COOH–Bn) and W3 (PO_3_H_2_–Bn) as having the most favorable donor–acceptor interactions among benzyl-substituted viologens, translating to enhanced thermodynamic stability. While phenyl analogues such as D3a (NH_2_–Ph) demonstrate even greater orbital delocalization, their high stabilization energies raise potential concerns related to over-delocalization. Therefore, the incorporation of moderately electron-withdrawing substituents, such as those in W2a (COOH–Ph) and W3a (PO_3_H_2_–Ph), offers a promising strategy for optimizing electronic delocalization while maintaining redox and structural integrity in asymmetric viologens.

### 2.2. Optical Behavior of Asymmetric Viologens

The optical behavior of asymmetric viologens across three oxidation states (e.g., dicationic, radical cationic and neutral) is governed by the nature of terminal substituents and the degree of conjugation in the molecular backbone. The central –CH_2_– linker contributes electron-donating character via hyperconjugation, thereby elevating the HOMO energy of donor fragments, which significantly influences the observed redox-dependent absorption properties [26].

**Figure 2:**
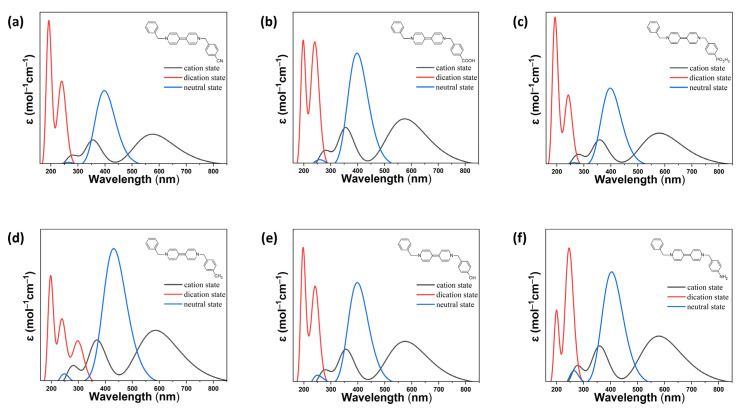
Simulated UV-Vis absorption spectra of the designed Bn-V-Bn viologen derivatives: (**a**) W1 (Bn-V-Bn-CN); (**b**) W2 (Bn-V-Bn-COOH); (**c**) W3 (Bn-V-Bn-PO_3_H_2_); (**d**) D1 (Bn-V-Bn-CH_3_); (**e**) D2 (Bn-V-Bn-OH); and (**f**) D3 (Bn-V-Bn-NH_2_). The neutral structure of each derivative is shown as an inset.

**Figure 3:**
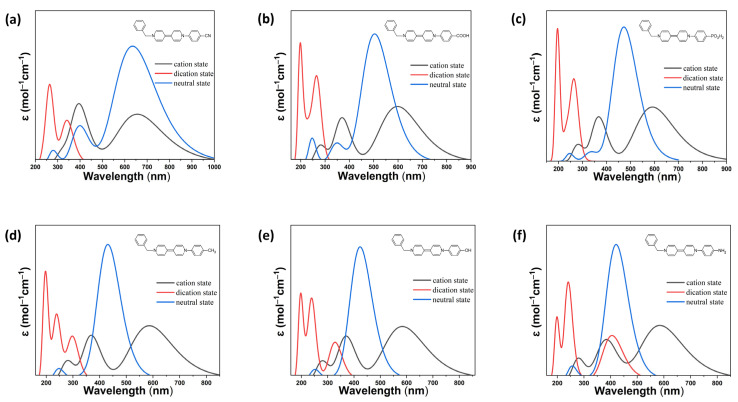
Simulated UV-Vis absorption spectra of the designed Bn-V-Ph viologen derivatives: (**a**) W1a (Bn-V-Ph-CN); (**b**) W2a (Bn-V-Ph-COOH); (**c**) W3a (Bn-V-Ph-PO_3_H_2_); (**d**) D1a (Bn-V-Ph-CH_3_); (**e**) D2a (Bn-V-Ph-OH); and (**f**) D3a (Bn-V-Ph-NH_2_). The neutral structure of each derivative is shown as an inset.

Calculated absorption data for all species are summarized in Table 2 (dicationic state), Table S13 (radical cationic state), and Table S14 (neutral state), while Figure 2 and Figure 3 visualize these trends for representative benzyl- and phenyl-substituted derivatives, respectively. The complementary use of numerical data and spectral profiles allows for a comprehensive understanding of how electronic structure modulates redox-responsive optical properties.

In the fully oxidized dicationic state, all viologen derivatives exhibit strong π→ π* transitions localized in the ultraviolet region. The precise absorption onset and intensity are dictated by the electron-donating or -withdrawing nature of the substituents.

As summarized in Table 2, electron-withdrawing substituents (W-series and W-a series), such as –CN, –COOH, and –PO_3_H_2_, yield dications with large HOMO–LUMO gaps (6.29–6.48 eV) and strong absorption in the deep UV (187–279 nm), with oscillator strengths ranging from moderate to high (*f* = 0.25–1.00). These transitions arise from frontier and near-frontier π-orbital interactions (e.g., H–2→ L, H→ L+5), indicating relatively localized excitations across the bipyridyl–substituent π-system.

Electron-donating groups (D-series and D-a series), such as –CH_3_, –OH, and –NH_2_, reduce the bandgap (down to ~4.94 eV for D3^2+^), red-shift the absorption onset, and increase charge-transfer character by destabilizing the HOMO. These species show that transitions extending closer to the visible region are still primarily governed by π→ π* interactions. Notably, amino-substituted D3^2+^ and D3a^2+^ exhibit the strongest red-shifts and highest oscillator strengths, reflecting enhanced delocalization and intramolecular charge transfer (ICT) [27].

The visual data in Figure 2 and Figure 3 support these trends. For example, W1 and W1a (–CN) show sharp, narrow absorption bands in the far-UV region, while D3 and D3a (–NH_2_) exhibit broader absorption features that approach the UV–visible boundary. The contrast between benzyl- and phenyl-terminated systems further illustrates the role of conjugation: the phenyl group introduces greater planarity and electronic coupling, resulting in slightly red-shifted and more structured UV absorption bands compared to their benzyl analogs.

One-electron reduction in the dications produces stable viologen radical cations, which exhibit distinct absorption features in the visible region. As shown in Table S13, all radical species display a dominant absorption band between 570 and 600 nm, corresponding to spin-allowed HOMO→LUMO transitions. These transitions typically involve >90% single-orbital contributions, with high oscillator strengths, indicating strongly allowed electronic excitations that play a central role in the electrochromic behavior of viologens. Secondary absorption bands in the 350–400 nm region, with moderate oscillator strengths (*f* = 0.4–0.6), arise from additional transitions involving deeper HOMOs (H − n) or higher LUMOs (L + m), indicating minor but non-negligible contributions from surrounding orbitals.

Figure 2 and Figure 3 show that radical absorption is relatively conserved across substitution patterns, but subtle differences emerge with donor strength and backbone rigidity. For instance, D2a and D3a exhibit slightly broader and red-shifted radical bands compared to W-series analogs, suggesting enhanced spin delocalization and greater orbital overlap in donor-rich systems. Similarly, phenyl-substituted radicals (e.g., D3a, W3a) display more intense and structured visible absorption than benzyl-substituted ones, reflecting the influence of extended conjugation and increased planarity on the radical state’s electronic configuration. These radical absorption features are essential for electrochromic applications, as they correspond to reversible color changes upon redox cycling. The distinct, intense, and substituent-sensitive bands in the visible region enable color tuning via molecular design.

Upon full reduction, the viologen derivatives form neutral species with significantly red-shifted absorption features, often extending deep into the visible region. As reported in Table S14, the extent of this red-shift depends strongly on the substituent’s electronic character and the conjugation length of the molecular backbone.

Electron-withdrawing groups, while stabilizing the LUMO, generally yield neutral species with absorption maxima around 370–400 nm and moderate oscillator strengths. In contrast, electron-donating groups promote stronger and more extended charge-transfer transitions, with intense absorption bands reaching beyond 450 nm. Notably, D3 and D3a (–NH_2_) exhibit the most prominent red-shifts and absorption intensities among all neutral species, consistent with their strong donor character and HOMO destabilization.

Figure 2 and Figure 3 further illustrate these distinctions. In the benzyl series (Figure 2), the neutral forms of D2 and D3 show clear bathochromic shifts compared to W1–W3, with visible absorption features that reflect substantial intramolecular ICT. In the phenyl series (Figure 3), this trend is even more pronounced. D3a, for instance, displays a broad and intense absorption band reaching up to ~550 nm, surpassing its benzyl counterpart and highlighting the synergistic effect of donor substitution and backbone conjugation.

Interestingly, the phenyl-substituted W-a derivatives (e.g., W1a–W3a) also show extended absorption in the visible range upon full reduction, though to a lesser degree than donor-substituted compounds. This suggests that even electron-withdrawing groups can support low-energy transitions when embedded in a conjugated, planar framework.

Overall, these results demonstrate that the optical properties of asymmetric viologens are highly tunable through deliberate substituent selection and backbone engineering. Electron-withdrawing groups (–CN, –COOH, –PO_3_H_2_) preserve large bandgaps and promote high-energy π→ π* transitions in the dicationic state, with moderate visible absorption in reduced forms unless conjugation is extended. In contrast, electron-donating substituents (–CH_3_, –OH, –NH_2_) induce pronounced red-shifts across all redox states by raising HOMO energies and enhancing intramolecular charge-transfer character, particularly in the radical and neutral species. These effects are further amplified in the phenyl-substituted series, where increased conjugation and molecular planarity stabilize delocalized excited states and improve absorption in the visible region.

**Figure 4:**
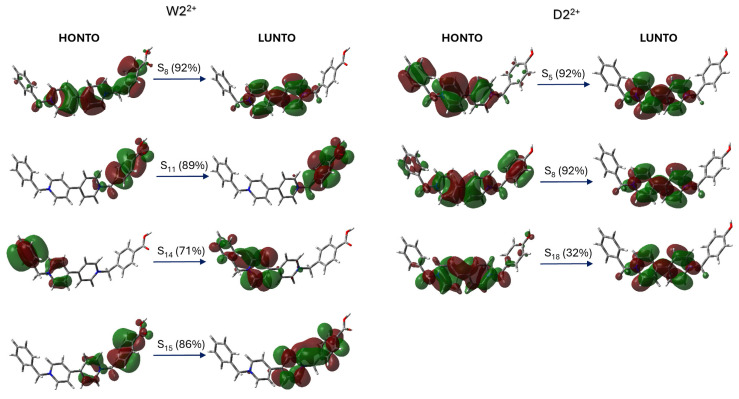
Natural Transition Orbital (NTO) analysis illustrating the calculated electronic excitations of W2^2+^ (Bn-V-Bn-COOH) and D2^2+^ (Bn-V-Bn-OH). For each complex, the dominant hole (HONTO) and electron (LUNTO) orbitals are shown for specific excitations S_n_, with corresponding transition contributions (%) indicated.

To accurately describe the observed electronic transitions, natural transition orbitals (NTOs) were computed using time-dependent density functional theory (TD-DFT) based on optimized geometries. These calculations identify the highest occupied natural transition orbital (HONTO) and the lowest unoccupied natural transition orbital (LUNTO), offering insights into the nature of the excitations. Figure 4 presents the NTOs for two representative systems, W2^2+^ (Bn-V-Bn-COOH) and D2^2+^ (Bn-V-Bn-OH, which exemplify the effects of electron-withdrawing and electron-donating substituents, respectively. The complete set of NTOs for the remaining molecules is provided in Table S15 of the Supplementary Information.

The depicted frontier orbitals in Figure 4 highlight how substituent electronics modulate charge distribution upon excitation. In the W2^2+^ system, the HONTO is primarily localized on the carboxyl-functionalized benzyl group, with minor contributions from the adjacent benzyl ring. The LUNTO extends over the carboxyl group and the bipyridinium core, indicating a pronounced charge transfer toward the central viologen unit. In contrast, the D2^2+^ system exhibits a more delocalized HONTO spanning both benzyl substituents and the bipyridinium moiety, while the LUNTO remains largely confined to the bipyridyl core with modest redistribution.

These results underscore the distinct impact of EWG and EDG substituents on the electronic structure of asymmetric viologens. Electron-withdrawing groups promote intramolecular charge transfer by directing electron density toward the viologen core, whereas electron-donating groups facilitate broader delocalization and diminish localized charge-transfer features [28]. These substituent-dependent orbital interactions are critical considerations in the design of viologen-based electrochromic and optoelectronic materials, as they influence key properties such as photoinduced charge separation efficiency, absorption characteristics, and color modulation behavior.

The computed electronic circular dichroism (ECD) spectra of a series of asymmetric viologen derivatives, specifically Bn-V-Bn and Bn-V-Ph frameworks bearing electron-withdrawing or electron-donating substituents, were analyzed across three oxidation states (neutral, monoradical cation, and dication) to investigate their chiroptical behavior in the context of designing redox-tunable electrochromic materials [29]. As shown in Figures S1 and S2 (Supplementary Information), these ECD profiles reveal distinct Cotton effects that serve as key spectroscopic signatures of asymmetric electronic transitions, offering critical insight into how molecular chirality and redox state interplay to modulate optical activity. The Cotton effect, representing the differential absorption of left- and right-circularly polarized light near electronic transitions, is particularly valuable in this context as it provides a direct probe of the chiral perturbation of excited states, making it a powerful diagnostic and design parameter for electrochromic systems with asymmetric optical outputs [29,30,31,32].

In the Bn-V-Bn series (Figure S1), all neutral species exhibit negligible absorption in the UV-visible region, indicating an absence of significant electronic transitions or intramolecular charge-transfer processes in their ground state [31]. Upon oxidation, dicationic species consistently display pronounced Cotton effects, while the monoradical cationic states remain nearly ECD-inactive, highlighting the strong redox dependence of chiral electronic responses.

Among electron-withdrawing substituents, the dicationic forms of W1 (CN), W2 (COOH), and W3 (PO_3_H_2_) exhibit intense negative Cotton effects primarily between ~190–230 nm. W1 features two sharp negative bands (~190 and ~220 nm, Δε ≈ −220 L mol^−1^ cm^−1^), attributable to the strong electron-withdrawing nature of the cyano group. W2 shows a broader negative band at ~230 nm (Δε ≈ −20) and weaker bisignate features between 180–200 nm, suggesting exciton-type coupling influenced by the carboxyl group. Similarly, W3 displays a strong negative band at ~190 nm (Δε = −100) and a shoulder at ~220 nm, consistent with electronic polarization induced by the phosphonic acid moiety.

In contrast, electron-donating substituents lead to distinct chiroptical behavior. D1 (CH_3_) exhibits a single intense positive Cotton effect at ~180 nm (Δε > +300), driven by hyperconjugation and inductive donation. D2 (OH) shows moderate negative bands at ~190 and ~210 nm (Δε = −15 to −35), likely stabilized by intramolecular hydrogen bonding, while D3 (NH_2_) presents strong negative Cotton effects at ~190 and ~230 nm (Δε ≈ −200), with minor positive features, indicating resonance-driven electron donation.

The Bn-V-Ph series (Figure S2) introduces phenyl bridges, which extend π-conjugation and significantly broaden the chiroptical activity across the 150–500 nm range. Unlike the Bn-V-Bn analogues, the neutral species in this series exhibit exciton-coupled circular dichroism signals characterized by a pair of oppositely signed Cotton effects, typically observed between 300–450 nm, arising from through-space interactions enabled by the phenylene spacer.

For electron-withdrawing derivatives (W1a–W3a), dicationic forms consistently show Cotton effects near 200–240 nm with alternating signs (e.g., W1a: +45 at ~200 nm, −40 at ~230 nm), alongside neutral-state bisignate bands (300–450 nm), pointing to enhanced delocalization and polarizability. Similarly, donor-substituted analogues (D1a–D3a) display dicationic Cotton effects between ~190–230 nm with moderate intensity (Δε ±15 to ±25), while the neutral forms show prominent bisignate features in the extended UV-visible region, indicating enhanced asymmetry via conjugative and hydrogen-bonding effects.

In summary, both series demonstrate that redox state, substituent electronics, and π-conjugation length collectively dictate the intensity, sign, and spectral position of Cotton effects. The presence or absence of exciton-type coupling, the electron-donating/withdrawing nature of substituents, and the extent of electronic delocalization are key parameters in tuning chiroptical responses. These findings affirm the utility of ECD spectroscopy as a predictive and tunable descriptor for designing redox-switchable chiroptical systems, with potential applications in circularly polarized displays, dynamic optical switches, and molecular information storage [33,34].

### 2.3. EC Properties

The redox potentials of the asymmetric viologen (AV) compounds were estimated using a thermodynamic cycle that combines gas-phase free energy changes with solvation effects. This computational approach is widely employed in quantum chemical studies of redox processes in solution. In this framework, the solvation free energies of the neutral molecule (AV^0^), the radical cation (AV^•+^), and the dication (AV^2+^) are represented as Δ*G_soln_*(AV^0^), Δ*G_soln_*(AV^•+^), and Δ*G_soln_*(AV^2+^), respectively. The intrinsic gas-phase redox free energy, Δ*G_gas_*, is evaluated from standard-state thermochemical data and quantum chemical calculations. These quantities are combined to obtain the overall Gibbs free energy change for each redox transformation in solution:

AV^0^ → AV^•+^ → AV^2+^

The solution-phase Gibbs free energy change associated with each redox step is calculated using the following expression:

(1) $$\Delta G_{soln}=\Delta G_{gas}+\left[\Delta E_{solv}-\Delta E_{gas}\right]+\Delta G_{corr}\;\;$$

where $\Delta E_{solv}$ and $\Delta E_{gas}$ are the single-point electronic energies calculated in solvent and gas phase, respectively. $\Delta G_{corr}$ accounts for the thermal correction and standard-state adjustment (from 1 atm to 1 M at 298 K), as well as any additional thermal contributions not captured by implicit solvation models.

The absolute reduction/oxidation potential ($E_{abs}^{o}$) is defined by the relation:

(2) $$E_{abs}^{o}=\frac{-\Delta G_{soln}}{nF}\;\;\;$$

where n is the number of electrons transferred in the redox process (here, n = 1), and F is the Faraday constant. The computed absolute redox potential is typically referenced against a standard electrode to enable comparison with experimental values. This referenced (or relative) standard reduction potential ($E^{o}$) is determined as:

(3) $$E^{o}=E_{abs}^{o}-E_{ref}^{o}\;\;$$

In this study, the Ag/AgCl reference electrode was used, with an assigned absolute potential of 4.90 V. The choice of reference electrode is critical to ensure consistency with experimental electrochemical measurements.

To evaluate the thermodynamic stability of intermediate radical species formed during redox cycling, the comproportionation constant ($K_{c}$) was calculated using the expression [35]:

(4) $$K_{c}=\exp\left(\frac{\Delta E_{red}}{25.69\;mV}\right)\;\;\;$$

where $\Delta E_{red}$ represents the difference between the first and second reduction potentials. A higher $K_{c}$ value indicates greater stability of the intermediate species.

Based on the computed redox potentials, clear trends emerge regarding how electron-donating and electron-withdrawing substituents influence the redox behavior of the asymmetric viologen compounds. The first (*E*1*_red_*) and second (*E*2*_red_*) reduction potentials, as well as the potential separation (Δ*E_red_*) and the corresponding comproportionation constant, are summarized in Table 3. *E*1*_red_* reflects the ease of converting the dication (AV^2+^) to the radical cation (AV^•+^), while *E*2*_red_* describes further reduction to the neutral species (AV^0^). In general, AV systems incorporating electron-withdrawing groups (W-series) exhibit more positive *E*1*_red_* values, indicating greater dication stabilization and enhanced reduction accessibility. Conversely, electron-donating substituents (D-series) shift *E*1*_red_* to more negative values, signifying increased difficulty in the initial reduction step but stronger stabilization of the resulting radical cation.

The estimated redox parameters show distinct trends in how substituents modulate the thermodynamic stability of the redox states. In the W-series, where electron-withdrawing groups are present, the first reduction potentials are generally less negative compared to those in the D-series, suggesting that the dicationic state is better stabilized and more readily reduced. For instance, W1 (Bn–V–Bn–CN) and W1a (Bn–V–Ph–CN) display relatively high *E*1*_red_* values due to the strong electron-withdrawing effect of the cyano group, which supports delocalization and charge stabilization upon reduction.

Additionally, W3 exhibits a significant potential separation (Δ*E_red_* = 0.84 V) and a high comproportionation constant (*K_c_* = 1.58 × 10^6^), indicating a thermodynamically stable radical cation intermediate. These characteristics are advantageous for applications involving mixed-valence or redox-switching behavior, such as in electrochromic devices or charge-storage materials.

In contrast, the D-series compounds, functionalized with electron-donating groups, display more negative *E*1*_red_* values, reflecting a reduced tendency for the dication to accept an electron. However, this is offset by greater radical cation stabilization, as evidenced by their larger Δ*E_red_* values. Among these, D3 shows the highest Δ*E_red_* (0.92 V) and *K_c_* (5.74 × 10^6^), signifying exceptional stability of the intermediate AV^•+^ species and strong thermodynamic preference for the comproportionated state.

The substituted phenyl-linked derivatives (W1a–D3a) follow similar electronic trends as their benzyl counterparts, though the introduction of the phenyl spacer influences conjugation and resonance effects. Notably, D1a, D2a, and D3a exhibit high Δ*E_red_* values and *K_c_* constants (e.g., D3a: 0.89 V, 3.67 × 10^6^), confirming that strong donor groups continue to support radical stabilization even when electronic communication is extended through a phenyl linkage.

Overall, the computational results highlight D3 and D3a (featuring NH_2_ substituents) as particularly promising candidates for electrochromic device applications due to their favorable balance of redox accessibility and outstanding radical cation stability. D2a and D1a also demonstrate desirable properties, benefiting from both extended conjugation and strong thermodynamic support for the intermediate species. These findings reinforce the importance of substituent tuning and molecular architecture in designing viologen-based systems for redox-active materials.

**Figure 5:**
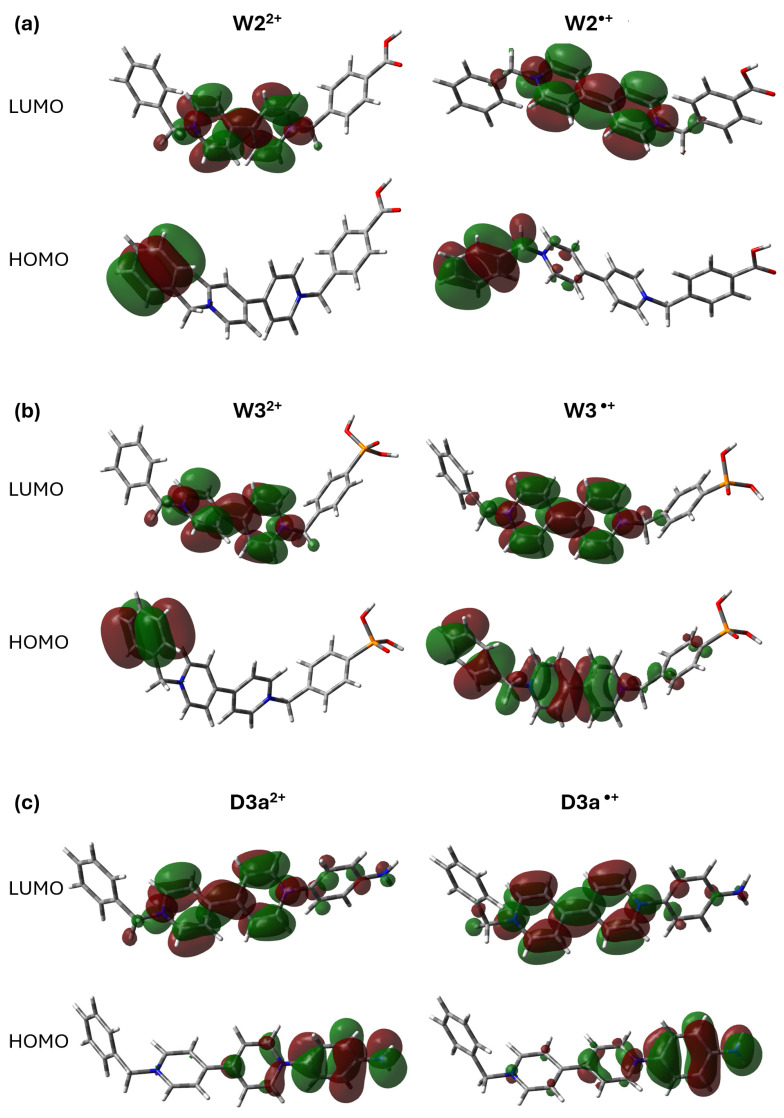
HOMO–LUMO molecular orbital diagrams of representative asymmetric viologens: (**a**) W2 (Bn–COOH), (**b**) W3 (Bn–PO_3_H_2_), and (**c**) D3a (Ph–NH_2_). Isosurfaces represent the frontier orbitals computed at the CAM-B3LYP/6-31+G(d,p) level.

The combined structural, electronic, optical, and NBO analyses establish a clear hierarchy of stability and functional suitability among the twelve asymmetric viologens studied. Benzyl-substituted compounds with strong electron-withdrawing groups, particularly W2 (Bn–COOH) and W3 (Bn–PO_3_H_2_), demonstrate consistently favorable characteristics. As shown in Figure 5, the HOMO–LUMO MO diagrams reveal large energy gaps and localized frontier orbitals, supporting high oxidative stability and effective charge separation. These compounds maintain structural rigidity and exhibit beneficial NBO charge delocalization, which enhances electron stabilization without causing excessive geometric distortion. Their functional groups (–COOH and –PO_3_H_2_) further facilitate electrode attachment and ion transport, ideal for electrochromic device applications. 

In contrast, the phenyl-substituted donor D3a (Ph–NH_2_) features more extended conjugation with a HOMO that is more delocalized across the molecule. This corresponds to a narrower HOMO–LUMO gap, consistent with stronger donor effects but also a risk of over-delocalization, which may impact electrochemical reversibility. These visual insights, combined with thermodynamic and NBO data, guide the design of viologen derivatives, balancing charge stabilization and redox performance. The HOMO–LUMO molecular orbital diagrams of the remaining molecules are provided in Figures S3–S11 of the Supplementary Information.

## 3. Methods and Materials

All computational studies were conducted using the Gaussian 16 Rev. C.01 software package [36]. The focus of this study is a series of asymmetric viologen derivatives with the general formula Bn–V–R, where Bn denotes a benzyl group attached to the viologen core, and R represents various electron-donating or electron-withdrawing substituents.

To ensure systematic analysis, each compound was assigned a specific code according to a standardized nomenclature scheme (Figure 1). Electron-withdrawing groups include CN (W1, W1a), COOH (W2, W2a), and PO_3_H_2_ (W3, W3a), while electron-donating groups include CH_3_ (D1, D1a), OH (D2, D2a), and NH_2_ (D3, D3a). The suffix “a” designates compounds where the substituent is located on a phenyl ring, while the unsubscripted codes refer to substitutions on the benzyl group.

Each derivative was studied in its neutral, radical cation, and dicationic oxidation states. Geometry optimizations were performed entirely in solvent using the Conductor-like Polarizable Continuum Model (C-PCM) with acetonitrile as the implicit solvent. All calculations employed density functional theory (DFT) using the CAM-B3LYP exchange correlation functional [37], a long-range corrected hybrid known for its reliability in modeling charge-transfer systems, along with the 6-31+G(d,p) basis set. The inclusion of diffuse and polarization functions in this basis set allows for more accurate treatment of charged and delocalized species.

The CAM-B3LYP functional was selected based on prior work by Jiang and colleagues [35] who demonstrated its effectiveness in capturing the electronic and redox behavior of viologen systems. This methodology is extended here by employing a more flexible basis set to improve the accuracy of predicted geometries and spectroscopic properties across a broader range of asymmetric viologens.

To further validate the choice of basis set, we performed a basis set convergence study using aug-cc-pVTZ as a high-accuracy reference. Two representative molecules were selected to reflect the range of electronic effects present in the series: W1^2+^, bearing an electron-withdrawing cyano group, and D2^2+^, bearing an electron-donating hydroxyl group. For the compounds W1^2+^ and D2^2+^, the mean absolute errors (MAEs) in vertical excitation energies, averaged over the first 20 singlet excited states, were found at 0.044 (W1^2+^) and 0.051 eV (D2^2+^), while the MAEs for oscillator strengths are at 0.087 (W1^2+^) and 0.069 (D2^2+^). These results fall within typical chemical accuracy limits [38,39], confirming that the 6-31+G(d,p) basis set provides reliable excitation energies and oscillator strengths for these systems. Given its favorable balance between computational cost and accuracy, 6-31+G(d,p) was selected for all reported calculations.

To ensure the reliability of the unrestricted DFT (UDFT) calculations used to model the radical cation species, the expectation values of the total spin-squared operator ⟨S^2^⟩ were evaluated after spin annihilation. All computed ⟨S^2^⟩ values were found to lie within a narrow range of 0.7501–0.7503, closely matching the ideal value of 0.75 for a pure doublet state. These results indicate negligible spin contamination and confirm the spin purity of the open-shell electronic wavefunctions used in this study.

No symmetry constraints were applied during geometry optimizations, allowing for complete structural relaxation. To confirm that all optimized structures correspond to true local minima on the potential energy surface, vibrational frequency analyses were performed at the same level of theory. The absence of imaginary frequencies verified the stability of each optimized structure.

To explore the electronic structure, Natural Bond Orbital (NBO) analysis was conducted on all species to assess atomic charges, charge delocalization, and bonding characteristics across different oxidation states. These data provided insights into how substituent electronics affect intramolecular charge distribution and redox behavior within the Bn–V–R framework.

Optical properties were investigated using time-dependent density functional theory (TD-DFT) at the CAM-B3LYP/6-31+G(d,p) level of theory, within the CPCM solvent model for acetonitrile, as implemented in Gaussian 16 [36]. Simulated UV-Vis absorption and electronic circular dichroism (ECD) spectra were obtained for the radical cation and dicationic forms of each compound. Full linear-response TD-DFT was used to compute excited-state properties, including absorption wavelengths (λ), oscillator strengths (*f*), and the nature of the electronic transitions. Natural Transition Orbitals (NTOs) were employed to provide a compact and chemically intuitive description of the excitations, based on the method described by R.L. Martin [40], where the transition density matrix is diagonalized to yield the dominant hole-particle orbital pairs. Additionally, HOMO–LUMO energy levels and energy gaps (Δ*E_HL_*) were computed for the dicationic species to evaluate how substituent effects modulate electronic structure. The leading NTO pairs and molecular orbital energy diagrams [41], representing the primary excitation character and electronic structure, respectively, were extracted from the TD-DFT results and visualized using GaussView 6.1.1 [42], enabling direct comparisons of EWG and EDG influences across the different Bn–V–R configurations.

## 4. Conclusions

A comprehensive DFT investigation was conducted on a series of twelve asymmetric viologen derivatives, each incorporating electron-donating or electron-withdrawing substituents at either the benzyl or phenyl termini. The results underscore the critical role of substituent identity and position in governing the structural, electronic, and optical properties of these redox-active systems.

Viologens bearing strong electron-withdrawing groups, such as Bn–COOH (W2) and Bn–PO_3_H_2_ (W3), exhibited pronounced planarity and minimal torsional strain across all oxidation states, promoting efficient charge delocalization and enhanced geometric stability. These derivatives also displayed wider HOMO–LUMO energy gaps, indicative of greater thermodynamic stability and reduced susceptibility to undesirable side reactions.

Optical analyses revealed sharp π→ π* transitions in the UV region and well-defined radical cation absorptions around 575–580 nm, consistent with reversible and distinguishable electrochromic behavior. Natural Bond Orbital (and second-order perturbation analyses further highlighted the role of intramolecular donor–acceptor interactions, with Bn–COOH and Bn–PO_3_H_2_ demonstrating balanced stabilization energies that facilitate charge redistribution without excessive delocalization. In contrast, donor-rich derivatives such as Bn–NH_2_ and Bn–OH exhibited narrower HOMO–LUMO gaps and reduced stabilization energies, potentially limiting their electrochemical durability despite offering tunable optical responses. Phenyl-substituted analogues, particularly Ph–NH_2_, showed increased orbital overlap and higher stabilization energies, though these strong donor effects may undermine long-term redox stability. Notably, Ph–COOH and Ph–PO_3_H_2_ provided a favorable balance between electronic stability and structural rigidity.

Collectively, these findings establish clear structure–property relationships for asymmetric viologens and offer a rational design strategy for optimizing electrochromic performance. Strategic selection of terminal substituents—particularly electron-withdrawing groups at the benzyl or phenyl position—can significantly enhance molecular stability, redox reversibility, and optical clarity. This work contributes valuable insights toward the development of next-generation viologen-based materials for high-performance and durable electrochromic applications.

## Figures and Tables

**Table 1:** Geometric parameters of the three redox states (dication, radical cation, and neutral) of asymmetric viologen Bn-V-R, calculated at the CAM-B3LYP/6-31+G(d,p) level of theory. Atom numbering is shown in the molecular structures included within the table.

Structures	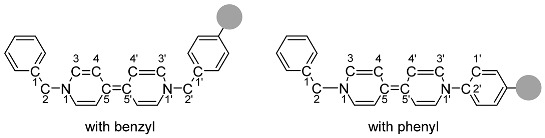					
	Bond Lengths (Å)			Torsion Angles (Degrees)		
	N1-C2	C5-C5′	N1′-C2′	C1-C2-N1-C3	C4-C5-C5′-C4′	C3′-N1′-C2′-C1′
(a) Neutral state						
W1	1.445	1.370	1.441	86.01	0.11	84.92
W2	1.444	1.370	1.442	85.81	0.10	85.36
W3	1.445	1.370	1.442	85.96	0.11	86.05
D1	1.444	1.370	1.445	85.62	0.05	85.63
D2	1.444	1.370	1.445	85.64	0.06	85.31
D3	1.444	1.371	1.454	85.85	0.23	60.05
W1a	1.447	1.373	1.395	86.88	1.30	29.38
W2a	1.447	1.373	1.398	86.84	1.30	29.80
W3a	1.447	1.373	1.398	86.86	0.64	30.24
D1a	1.445	1.372	1.411	86.10	0.54	36.92
D2a	1.445	1.371	1.413	86.10	0.91	40.62
D3a	1.444	1.371	1.415	86.07	0.47	42.33
(b) Radical cation state						
W1	1.467	1.424	1.464	88.46	0.08	88.52
W2	1.481	1.424	1.474	88.44	0.01	88.55
W3	1.481	1.424	1.475	49.92	0.15	56.26
D1	1.466	1.424	1.467	88.37	0.07	88.35
D2	1.466	1.424	1.467	88.41	0.05	88.53
D3	1.478	1.424	1.489	47.61	0.07	38.46
W1a	1.483	1.425	1.433	45.06	2.37	49.42
W2a	1.482	1.424	1.435	45.89	2.35	49.52
W3a	1.482	1.424	1.436	49.53	0.98	50.34
D1a	1.481	1.424	1.483	47.06	0.92	53.64
D2a	1.480	1.424	1.439	47.02	0.87	56.59
D3a	1.479	1.424	1.439	47.83	0.87	56.62
(c) Dicationic state						
W1	1.526	1.487	1.519	4.01	42.35	19.99
W2	1.526	1.487	1.519	4.18	42.29	23.39
W3	1.526	1.487	1.523	3.86	42.14	10.38
D1	1.525	1.487	1.528	3.45	42.43	0.04
D2	1.525	1.487	1.532	3.17	42.18	5.08
D3	1.524	1.487	1.541	5.84	42.17	1.55
W1a	1.527	1.487	1.457	1.07	41.88	56.74
W2a	1.526	1.487	1.457	2.12	41.78	55.40
W3a	1.526	1.487	1.457	3.54	42.26	55.48
D1a	1.525	1.486	1.454	4.85	41.86	52.60
D2a	1.525	1.486	1.449	1.18	40.75	50.60
D3a	1.524	1.484	1.440	3.87	39.94	45.06

**Table 2:** The absorption energies (λ in nm), oscillator strength (***f*** in a.u.), corresponding MO transitions, HOMO, LUMO energies in eV, and HOMO-LUMO gap (G_H-L_ in eV) for the dicationic state of asymmetric viologen derivatives calculated at CAM-B3LYP/6-31+G(d,p) level of theory in acetonitrile using C-CPM framework.

Cpds	State	λ	*f*	Transition Assignment (%)	HOMO	LUMO	G_H-L_
W1^2+^	S_0_ → S_5_	244	0.511	H-2 → L = 19	−9.01	−2.55	6.46
	S_0_ → S_7_	239	0.487	H-2 → L = 17			
	S_0_ → S_10_	227	0.515	H-2 → L+1 = 42			
	S_0_ → S_13_	199	0.658	H-1 → L+5 = 12; H → L+5 = 31			
	S_0_ → S_16_	194	0.841	H-3 → L+1 = 1; H-3 → L+6 = 22			
W2^2+^	S_0_ → S_8_	279	0.578	H-7 → L+1 = 29; H-2 → L = 11	−9.01	−2.54	6.47
	S_0_ → S_11_	239	0.487	H-3 → L+1 = 22; H-3 → L+2 = 20			
	S_0_ → S_14_	228	0.423	H-1 → L+5 = 14; H → L+5 = 28			
	S_0_ → S_15_	194	0.841	H-3 → L+3 = 16; H-3 → L+6 = 6; H-6 → L+3 = 1			
W3^2+^	S_0_ → S_4_	247	0.258	H-4 → L = 37; H-4 → L+1 = 2	−9.01	−2.53	6.48
	S_0_ → S_7_	239	0.487	H-7 → L = 25; H-2 → L+1 = 1			
	S_0_ → S_14_	239	0.474	H-2 → L+2 = 20; H-3 → L+4 = 5			
	S_0_ → S_19_	194	0.841	H-1 → L+8 = 26; H-1 → L+3 = 2			
D1^2+^	S_0_ → S_5_	247	0.265	H-4 → L = 39; H-1 → L = 3	−8.66	−2.49	6.17
	S_0_ → S_7_	239	0.487	H-6 → L = 41; H-4 → L = 2			
	S_0_ → S_15_	240	0.908	H-2 → L+2 = 20; H-2 → L+3 = 12			
	S_0_ → S_18_	194	0.841	H → L+7 = 26			
D2^2+^	S_0_ → S_5_	246	0.313	H-5 → L = 19; H-4 → L = 18; H-1 → L = 6	−8.24	−2.48	5.76
	S_0_ → S_8_	239	0.748	H-6 → L = 35; H-5 → L = 1			
	S_0_ → S_18_	198	0.687	H-1 → L+2 = 20; H-1 → L+3 = 13			
D3^2+^	S_0_ → S_6_	250	0.945	H → L+3 = 6; H → L+5 = 9; H → L+6 = 5	−7.42	−2.46	4.96
	S_0_ → S_11_	239	0.625	H-6 → L = 34; H → L+7 = 2			
	S_0_ → S_18_	200	0.532	H-1 → L+3 = 36; H-1 → L+8 = 8			
W1a^2+^	S_0_ → S_1_	267	0.841	H-6 → L = 5; H-2 → L = 27	−9.02	−2.73	6.29
	S_0_ → S_7_	234	0.307	H-6 → L = 39 H-2 → L+1 = 3			
	S_0_ → S_13_	199	0.592	H → L+3 = 21; H → L+4 = 10			
	S_0_ → S_18_	194	0.929	H-3 → L+2 = 22			
W2a^2+^	S_0_ → S_1_	269	1.001	H-3 → L = 33 H-1 → L = 2	−9.02	−2.70	6.32
	S_0_ → S_7_	234	0.345	H-7 → L = 40; H-3 → L = 2			
	S_0_ → S_14_	199	0.619	H → L+1 = 1; H → L+4 = 26			
	S_0_ → S_16_	196	0.570	H-3 → L+2 = 4; H-2 → L+2 = 26			
W3a^2+^	S_0_ → S_1_	268	0.953	H-3 → L+1 = 3; H-3 → L = 35; H-1 → L = 2	−9.02	−2.68	6.34
	S_0_ → S_7_	231	0.356	H-8 → L = 31; H-5 → L = 14; H-4 → L = 11			
	S_0_ → S_13_	199	0.642	H → L+3 = 31			
	S_0_ → S_17_	194	0.654	H-2 → L+2 = 27; H-3 → L+4 = 4			
	S_0_ → S_20_	188	0.305	H-4 → L+1 = 11; H → L+8 = 7			
D1a^2+^	S_0_ → S_1_	299	0.605	H-6 → L = 1; H → L = 43	−8.90	−2.64	6.26
	S_0_ → S_7_	237	0.698	H-6 → L = 44; H → L+1 = 1			
	S_0_ → S_15_	199	0.587	H-4 → L+3 = 2; H-2 → L+4 = 12; H-1 → L+3 = 30			
	S_0_ → S_19_	192	0.528	H-3 → L+6 = 18; H → L+5 = 15			
D2a^2+^	S_0_ → S_1_	328	0.561	H → L = 44; H → L+1 = 4	−8.44	−2.63	5.81
	S_0_ → S_8_	237	0.625	H-6 → L = 36; H → L+5 = 5			
	S_0_ → S_15_	199	0.667	H-2 → L+4 = 12; H-1 → L+2 = 4; H-1 → L+3 = 27			
D3a^2+^	S_0_ → S_1_	405	0.628	H → L = 44; H → L+1 = 4	−7.55	−2.61	4.94
	S_0_ → S_10_	240	0.937	H-7 → L = 4; H-6 → L = 23; H-4 → L = 16			
	S_0_ → S_18_	198	0.684	H-2 → L+4 = 12; H-1 → L+2 = 19; H-1 → L+3 = 13			

**Table 3:** The calculated first (*E*1*_red_* in V) and second (*E*2*_red_* in V) potentials and the Comproportionation constant (*K_c_*) for the asymmetric viologen derivatives.

Compound	*E*1*_red_* (V)	*E*2*_red_* (V)	Δ*E_red_*	*K_c_*
W1	−0.66	−1.28	0.63	3.97 × 10^4^
W2	−0.68	−1.28	0.60	2.51 × 10^4^
W3	−0.57	−1.42	0.84	1.58 × 10^6^
D1	−0.82	−1.31	0.49	3.97 × 10^3^
D2	−0.76	−1.28	0.35	6.30 × 10^3^
D3	−0.60	−1.52	0.92	5.74 × 10^6^
W1a	−0.45	−1.23	0.78	4.98 × 10^5^
W2a	−0.47	−1.26	0.79	6.22 × 10^5^
W3a	−0.55	−1.09	0.54	9.98 × 10^3^
D1a	−0.53	−1.39	0.85	1.91 × 10^6^
D2a	−0.53	−1.41	0.88	2.72 × 10^6^
D3a	−0.56	−1.45	0.89	3.67 × 10^6^

## Data Availability

The original contributions presented in the study are included in the article/Supplementary Materials; further inquiries can be directed to the corresponding authors.

## Supplementary Materials

The following supporting information can be downloaded at: https://www.mdpi.com/article/10.3390/ijms262010137/s1.
