# Supplementary material for: Computational Analysis of Electron-Donating and Withdrawing Effects on Asymmetric Viologens for Enhanced Electrochromic Performance
Source: Int J Mol Sci. 2025 Oct 18;26(20):10137. doi: 10.3390/ijms262010137 (PMC12564689; doi:10.3390/ijms262010137)
Supplement: Supplementary file 1 [file ijms-26-10137-s001.zip › ijms-3901006-supplementary.pdf]

International Journal of *Molecular Sciences*

MDPI

SUPPORTING INFORMATION

Computational Analysis of Electron-Donating and Withdrawing Effects on Asymmetric Viologens for Enhanced Electrochromic Performance

Gulzat Nuroldayeva[1, 2] and Mannix P. Balanay[1,2] *

[1] Department of Chemistry, Nazarbayev University, 53 Kabanbay Batyr Ave., Astana 010000, Kazakhstan; gulzat.nuroldayeva@nu.edu.kz

[2] National Laboratory of Astana, Nazarbayev University, 53 Kabanbay Batyr Ave., Astana 010000, Kazakhstan

* Correspondence: mannix.balanay@nu.edu.kz; Tel.: +7 7172 69 4657

Table S1. The atomic charges for the selected atoms of W1 $^{2+}$, W1 $^{+\bullet}$, and the charge differences calculated using the NBO method.

Atoms	System		Charge difference
	W1 $^{2+}$	W1 $^{+\bullet}$	ΔQ
N1	-0.294	-0.379	-0.085
C2	0.085	0.030	-0.055
C3	-0.234	-0.259	-0.025
C4	0.023	-0.049	-0.072
C6	0.092	0.041	-0.051
C7	-0.276	-0.276	0
C9	-0.226	-0.235	-0.009
C10	-0.187	-0.177	0.01
C11	-0.125	-0.161	-0.036
N13	-0.226	-0.275	-0.049

Table S2. The atomic charges for the selected atoms of W2 $^{2+}$, W2 $^{+\bullet}$, and the charge differences calculated using the NBO method.

Atoms	System		Charge difference
	W2 $^{2+}$	W2 $^{+\bullet}$	ΔQ
N1	-0.293	-0.375	-0.082
C2	0.085	0.043	-0.042
C3	-0.235	-0.266	-0.031
C4	0.021	0.048	0.027
C6	0.094	0.032	-0.062
C7	-0.275	-0.275	0
C9	-0.230	-0.243	-0.013
C10	-0.180	-0.174	0.006
C11	-0.130	-0.166	-0.036
O13	-0.727	-0.735	-0.008

Table S3. The atomic charges for the selected atoms of W3 $^{2+}$, W3 $^{+•}$, and the charge differences calculated using the NBO method.

Atoms	System		Charge difference
	W3 $^{2+}$	W3 $^{+•}$	ΔQ
N1	-0.294	-0.321	-0.027
C2	0.084	0.086	0.002
C3	-0.235	-0.249	-0.014
C4	0.022	0.070	0.048
C6	0.093	0.091	-0.002
C7	-0.275	-0.266	0.009
C9	-0.226	-0.247	-0.021
C10	-0.190	-0.228	-0.038
C11	-0.435	-0.408	0.027
P12	2.375	2.431	0.056
O13	-1.029	-1.051	-0.022

Table S4. The atomic charges for the selected atoms of D1 $^{2+}$, D1 $^{+•}$, and the charge differences calculated using the NBO method.

Atoms	System		Charge difference
	D1 $^{2+}$	D1 $^{+•}$	ΔQ
N1	-0.293	-0.366	-0.073
C2	0.082	0.035	-0.047
C3	-0.236	-0.261	-0.025
C4	0.017	-0.046	-0.063
C6	0.095	0.035	-0.06
C7	-0.273	-0.270	0.003
C9	-0.217	-0.245	-0.028
C10	-0.227	-0.226	0.001
C11	0.015	-0.024	-0.039
C12	-0.733	-0.722	0.011

Table S5. The atomic charges for the selected atoms of D2 $^{2+}$, D2 $^{+•}$, and the charge differences calculated using the NBO method.

Atoms	System		Charge difference
	D2 $^{2+}$	D2 $^{+•}$	ΔQ
N1	-0.294	-0.367	-0.073
C2	0.082	0.035	-0.047
C3	-0.236	-0.266	-0.03
C4	0.016	-0.024	-0.04
C6	0.094	0.035	-0.059
C7	-0.272	-0.267	0.005
C9	-0.206	-0.217	-0.011
C10	-0.273	-0.268	0.005
C11	0.361	0.328	-0.033
O12	-0.687	-0.706	-0.019

Table S6. The atomic charges for the selected atoms of D3 $^{2+}$, D3 $^{+\bullet}$, and the charge differences calculated using the NBO method.

Atoms	System		Charge difference
	D3 $^{2+}$	D3 $^{+\bullet}$	ΔQ
N1	-0.294	-0.373	-0.079
C2	0.081	0.042	-0.039
C3	-0.236	-0.271	-0.035
C4	0.013	0.024	0.011
C6	0.094	0.048	-0.046
C7	-0.270	-0.267	0.003
C9	-0.197	-0.201	-0.004
C10	-0.287	-0.283	0.004
C11	0.214	0.19	-0.024
N12	-0.845	-0.867	-0.022

Table S7. The atomic charges for the selected atoms of W1a $^{2+}$, W1a $^{+\bullet}$, and the charge differences calculated using the NBO method.

Atoms	System		Charge difference
	W1a $^{2+}$	W1a $^{+\bullet}$	ΔQ
N1	-0.290	-0.380	-0.09
C2	0.092	0.038	-0.054
C3	-0.235	-0.266	-0.031
C4	0.027	-0.031	-0.058
C6	0.092	0.038	-0.054
C7	0.097	0.144	0.047
C8	-0.241	-0.248	-0.007
C9	-0.168	-0.175	-0.007
C10	-0.113	-0.152	-0.039
C11	0.243	0.267	0.024
N12	-0.205	-0.261	-0.056

Table S8. The atomic charges for the selected atoms of W2a $^{2+}$, W2a $^{+\bullet}$, and the charge differences calculated using the NBO method.

Atoms	System		Charge difference
	W2a $^{2+}$	W2a $^{+\bullet}$	ΔQ
N1	-0.287	-0.376	-0.089
C2	0.092	0.040	-0.052
C3	-0.236	-0.268	-0.032
C4	0.023	-0.029	-0.052
C6	0.092	0.041	-0.051
C7	0.098	0.144	0.046
C8	-0.248	-0.256	-0.008
C9	-0.176	-0.181	-0.005
C10	-0.118	-0.156	-0.038
C11	0.812	0.816	0.004
O12	-0.725	-0.731	-0.006

Table S9. The atomic charges for the selected atoms of W3a $^{2+}$, W3a $^{+•}$, and the charge differences calculated using the NBO method.

Atoms	System		Charge difference
	W3a $^{2+}$	W3a $^{+•}$	ΔQ
N1	-0.288	-0.371	-0.083
C2	0.092	0.044	-0.048
C3	-0.236	-0.265	-0.029
C4	0.025	-0.029	-0.054
C6	0.092	0.044	-0.048
C7	0.100	0.142	0.042
C8	-0.246	-0.262	-0.016
C9	-0.175	-0.187	-0.012
C10	-0.423	-0.438	-0.015
P11	2.374	2.389	0.015
O12	-1.032	-1.047	-0.015

Table S10. The atomic charges for the selected atoms of D1a $^{2+}$, D1a $^{+•}$, and the charge differences calculated using the NBO method.

Atoms	System		Charge difference
	D1a $^{2+}$	D1a $^{+•}$	ΔQ
N1	-0.280	-0.369	-0.089
C2	0.088	0.044	-0.044
C3	-0.235	-0.27	-0.035
C4	0.016	-0.027	-0.043
C6	0.089	0.044	-0.045
C7	0.071	0.106	0.035
C8	-0.239	-0.242	-0.003
C9	-0.211	-0.221	-0.01
C10	0.025	-0.011	-0.036
C11	-0.735	-0.725	0.01

Table S11. The atomic charges for the selected atoms of D2a $^{2+}$, D2a $^{+•}$, and the charge differences calculated using the NBO method.

Atoms	System		Charge difference
	D2a $^{2+}$	D2a $^{+•}$	ΔQ
N1	-0.277	-0.361	-0.084
C2	0.084	0.033	-0.051
C3	-0.234	-0.264	-0.03
C4	0.013	-0.027	-0.04
C6	0.085	0.033	-0.052
C7	0.050	0.082	0.032
C8	-0.222	-0.226	-0.004
C9	-0.292	-0.303	-0.0106
C10	0.370	0.338	-0.032
O11	-0.676	-0.700	-0.024

Table S12. The atomic charges for the selected atoms of D3a $^{2+}$, D3a $^{+\bullet}$, and the charge differences calculated using the NBO method.

Atoms	System		Charge difference
	D3a $^{2+}$	D3a $^{+\bullet}$	ΔQ
N1	-0.270	-0.356	-0.086
C2	0.074	0.035	-0.039
C3	-0.231	-0.265	-0.034
C4	-0.002	-0.027	-0.025
C6	0.074	0.035	-0.039
C7	0.035	0.063	0.028
C8	-0.220	-0.223	-0.003
C9	-0.270	-0.279	-0.009
C10	0.226	0.192	-0.034
N11	-0.824	-0.863	-0.039

Table S13. The absorption energies (λ in nm), oscillator strength (f in a.u.), corresponding MO transitions, HOMO, LUMO energies in eV, and HOMO-LUMO gap (G_{H-L} in eV) for the radical cationic state of asymmetric viologen derivatives calculated at CAM-B3LYP/6-31+G(d,p) level of theory in acetonitrile using C-CPM framework.

**Cpds**	**State**	**λ**	***f***	**Transition assignment (%)**	**HOMO**	**LUMO**	**G_{H-L}**
W1 $^{+\bullet}$	$S_0 \rightarrow S_1$	576	0.685	H→L+1 = 96	-8.575	-2.021	6.554
	$S_0 \rightarrow S_5$	356	0.555	H-7→L+4 = 2; H-3→L = 87			
W2 $^{+\bullet}$	$S_0 \rightarrow S_1$	575	0.685	H→L+1 = 96	-8.572	-1.996	6.576
	$S_0 \rightarrow S_5$	357	0.555	H-3→L = 87			
W3 $^{+\bullet}$	$S_0 \rightarrow S_1$	638	0.049	H-9→L = 52; H-7→L = 17	-8.646	-1.937	6.709
	$S_0 \rightarrow S_4$	507	0.026	H-9→L = 23; H-8→L = 9; H-3→L = 49			
	$S_0 \rightarrow S_{15}$	268	0.123	H-7→L+6 = 3; H-7→L+7 = 4			
D1 $^{+\bullet}$	$S_0 \rightarrow S_1$	575	0.682	H→L = 96	-8.231	-1.976	6.255
	$S_0 \rightarrow S_4$	357	0.540	H-7→L+3 = 2; H-5→L+1 = 2; H-4→L = 86			
D2 $^{+\bullet}$	$S_0 \rightarrow S_1$	575	0.682	H→L = 96	-7.871	-1.978	5.893
	$S_0 \rightarrow S_4$	357	0.542	H-7→L+2 = 2; H-5→L+1 = 2; H-4→L = 86			
D3 $^{+\bullet}$	$S_0 \rightarrow S_1$	579	0.686	H→L = 96	-7.302	-1.866	5.436
	$S_0 \rightarrow S_5$	360	0.546	H-4→L = 47; H→L = 4			
	$S_0 \rightarrow S_{12}$	288	0.140	H-6→L = 76			
W1a $^{+\bullet}$	$S_0 \rightarrow S_1$	598	0.832	H→L = 67	-8.654	-2.143	6.511
	$S_0 \rightarrow S_5$	372	0.623	H-4→L = 4; H→L = 41			
W2a $^{+\bullet}$	$S_0 \rightarrow S_1$	598	0.821	H→L = 62	-8.634	-2.114	6.520
	$S_0 \rightarrow S_5$	371	0.631	H-5→L = 8; H-5→L+1 = 3; H→L = 45			
W3a $^{+\bullet}$	$S_0 \rightarrow S_1$	592	0.799	H→L = 87; H→L+2 = 9	-8.642	-2.091	6.551
	$S_0 \rightarrow S_5$	364	0.402	H→L = 25; H-2→L = 10			
	$S_0 \rightarrow S_{11}$	293	0.134	H-6→L = 75; H-4→L = 4			
D1a $^{+\bullet}$	$S_0 \rightarrow S_1$	586	0.765	H→L = 95; H→L+5 = 1	-8.362	-1.996	6.366
	$S_0 \rightarrow S_4$	373	0.511	H→L = 53			

Continuation Table S13

Cpds	State	λ	*f*	Transition assignment (%)	HOMO	LUMO	G_{H-L}
D2a $^{+\bullet}$	$S_0 \to S_1$	584	0.754	H→L = 96	-8.089	-1.980	6.109
	$S_0 \to S_4$	375	0.512	H→L = 43; H-3→L = 18; H-4→L = 10			
	$S_0 \to S_{16}$	268	0.125	H-5→L+1 = 25; H-6→L+7 = 8			
D3a $^{+\bullet}$	$S_0 \to S_1$	585	0.769	H→L = 95	-7.371	-1.945	5.426
	$S_0 \to S_4$	395	0.433	H→L = 4; H-4→L = 18; H-1→L = 5			

Table S14. The absorption energies (λ in nm), oscillator strength (f in a.u.), corresponding MO transitions, HOMO, LUMO energies in eV, and HOMO-LUMO gap (G_{H-L} in eV) for the neutral state of asymmetric viologen derivatives calculated at CAM-B3LYP/6-31+G(d,p) level of theory in acetonitrile using C-CPM framework.

Cpds	State	λ	*f*	Transition assignment (%)	HOMO	LUMO	G_{H-L}
W1 º	$S_0 \to S_3$	399	1.546	H→L+3 = 3; H→L+4 = 28; H→L+8 = 3	-4.849	-0.445	4.404
	$S_0 \to S_5$	368	0.126	H→L+3 = 44			
W2 º	$S_0 \to S_3$	400	1.545	H→L+3 = 44	-4.836	-0.534	4.302
	$S_0 \to S_5$	369	0.129	H→L+3 = 3; H→L+4 = 29; H→L+9 = 3			
W3 º	$S_0 \to S_2$	400	1.542	H→L+4 = 44; H→L+5 = 3	-4.838	-0.064	4.774
	$S_0 \to S_4$	369	0.125	H→L+5 = 33; H→L+11 = 2			
D1 º	$S_0 \to S_2$	400	1.522	H→L+1 = 43	-4.802	0.438	5.240
	$S_0 \to S_4$	372	0.138	H→L+3 = 37; H→L+1 = 3			
D2 º	$S_0 \to S_2$	400	1.519	H→L+1 = 44	-5.245	0.439	5.684
	$S_0 \to S_4$	371	0.139	H→L+3 = 35; H→L+9 = 3			
	$S_0 \to S_{19}$	246	0.114	H-1→L+2 = 21			
D3 º	$S_0 \to S_2$	406	1.518	H→L+1 = 41	-4.792	0.471	5.263
	$S_0 \to S_4$	368	0.126	H→L+3 = 31; H→L+5 = 3			
W1a º	$S_0 \to S_1$	498	1.971	H→L = 46	-5.038	-0.554	4.484
	$S_0 \to S_5$	343	0.136	H→L+3 = 37; H→L+4 = 3; H→L+11 = 3			
	$S_0 \to S_{18}$	243	0.203	H-1→L = 41			
W2a º	$S_0 \to S_1$	504	1.883	H→L = 45; H→L+3 = 3	-5.013	-0.631	4.382
	$S_0 \to S_{16}$	250	0.179	H-1→L = 7; H→L+5 = 5; H→L+8 = 5			
	$S_0 \to S_{17}$	247	0.118	H-6→L+3 = 1; H-1→L = 34			
W3a º	$S_0 \to S_1$	473	1.981	H→L = 47	-5.007	-0.282	4.725
D1a º	$S_0 \to S_2$	431	1.933	H→L = 48	-4.635	0.375	5.010
D2a º	$S_0 \to S_2$	424	1.889	H→L = 48	-4.842	0.333	5.175
D3a º	$S_0 \to S_2$	422	1.919	H→L = 48	-4.518	0.547	5.065

Table S15. Natural transition orbital (NTO) analysis of representative excited states for asymmetric viologen dications.

Cpds	λ, nm	State (% contribution)	HONTO	LUNTO
$W1^{2+}$	244	S_5 (86%)	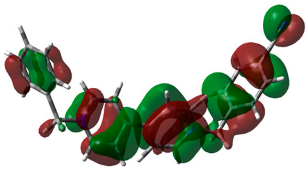	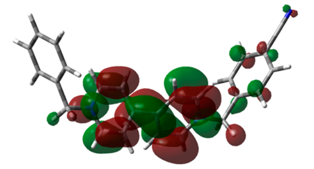
	239	S_7 (94%)	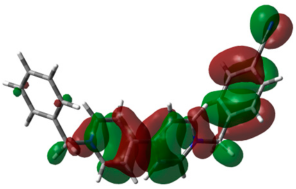	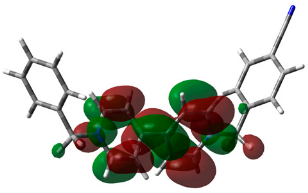
	227	S_{10} (89%)	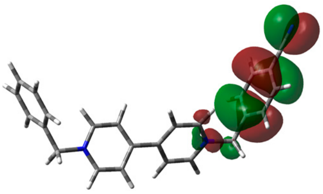	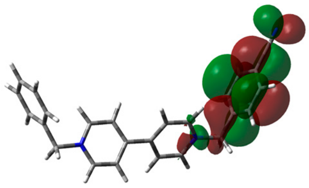
	199	S_{13} (73%)	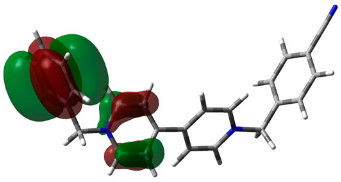	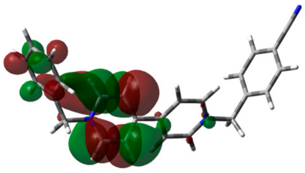
	194	S_{16} (86%)	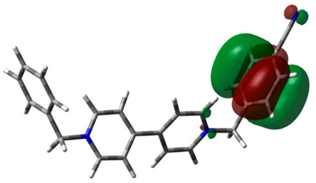	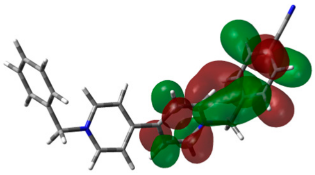
$W2^{2+}$	279	S_8 (92%)	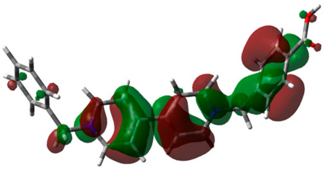	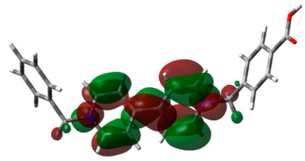
	239	S_{11} (89%)	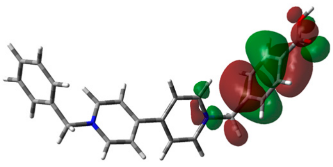	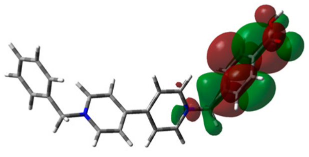
	228	S_{14} (71%)	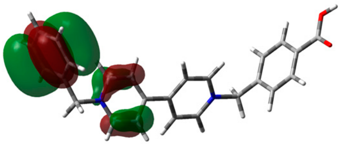	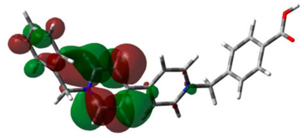

Continuation Table S15.

Cpds	λ, nm	State (% contribution)	HONTO	LUNTO
	194	S_{15} (86%)	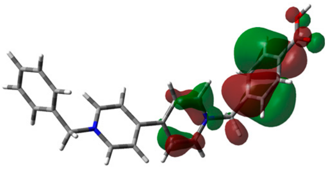	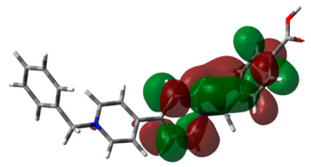
	247	S_4 (92%)	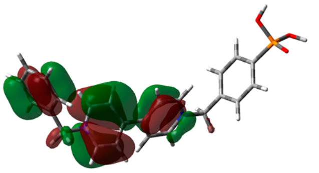	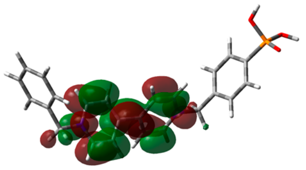
	239	S_7 (92%)	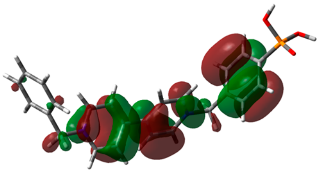	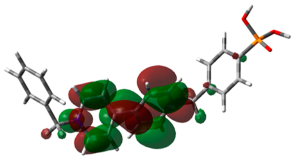
$W3^{2+}$				
	239	S_{14} (76%)	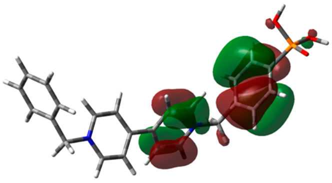	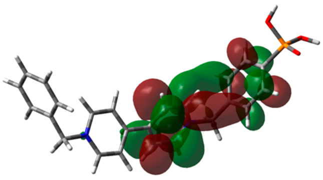
	194	S_{19} (67%)	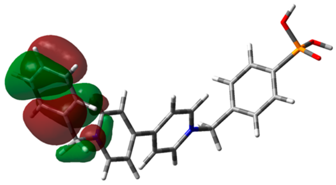	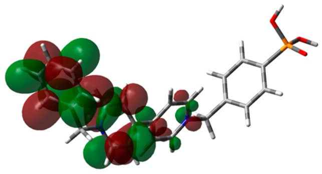
	247	S_5 (89%)	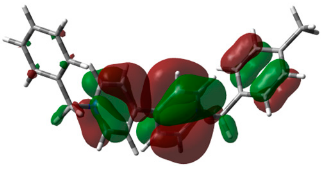	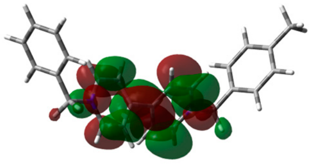
$D1^{2+}$	239	S_7 (94%)	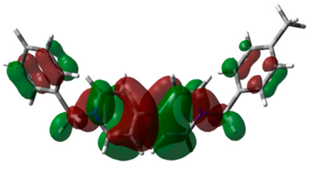	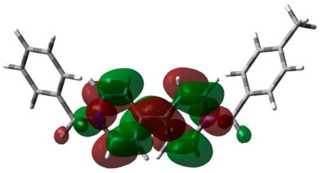
	240	S_{15} (72%)	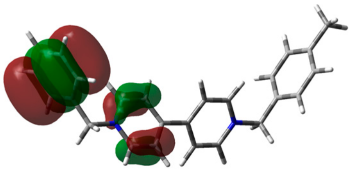	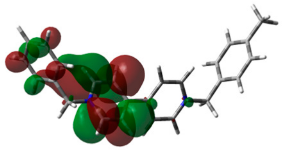

Continuation Table S15.

Cpds	λ, nm	State (% contribution)	HONTO	LUNTO
	194	S_{18} (58%)	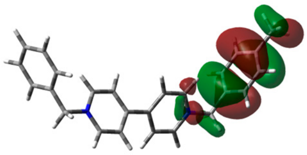	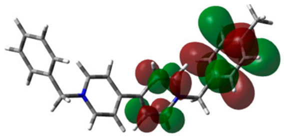
	246	S_5 (92%)	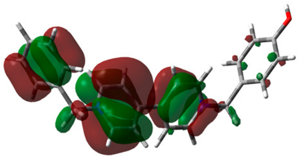	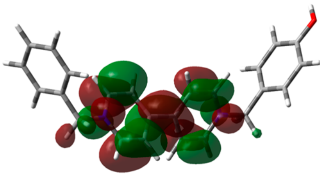
$D2^{2+}$	239	S_8 (92%)	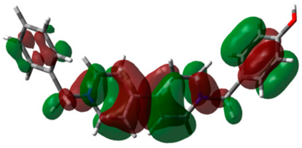	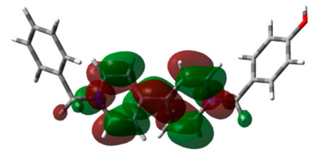
	198	S_{18} (32%)	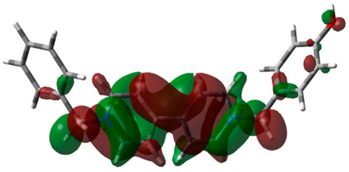	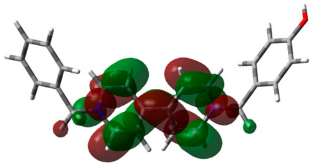
	250	S_6 (72%)	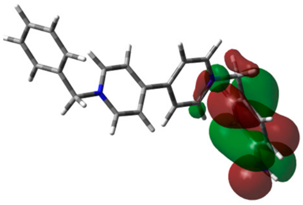	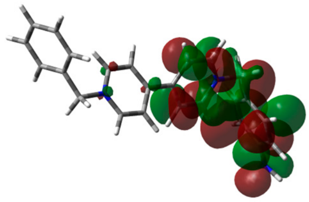
$D3^{2+}$	239	S_{11} (83%)	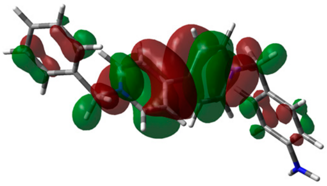	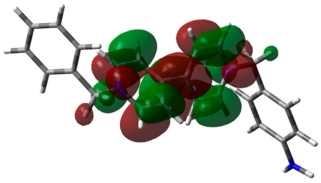
	200	S_{18} (92%)	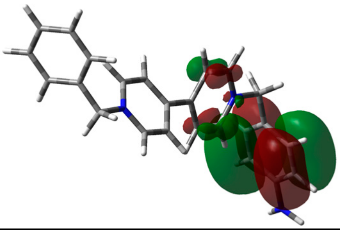	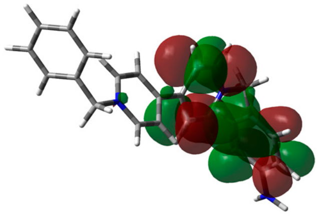
$W1a^{2+}$	267	S_1 (91%)	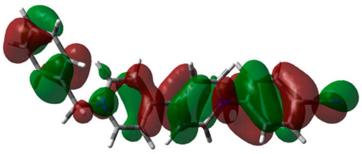	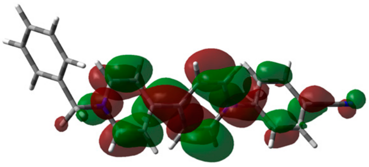

Continuation Table S15.

Cpds	λ, nm	State (% contribution)	HONTO	LUNTO
	234	S_7 (90%)	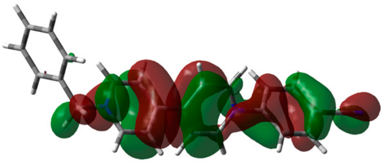	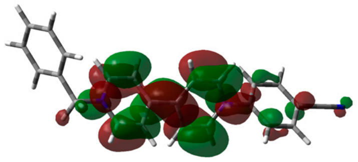
	199	S_{13} (73%)	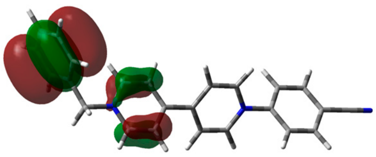	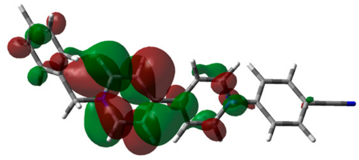
	194	S_{18} (85%)	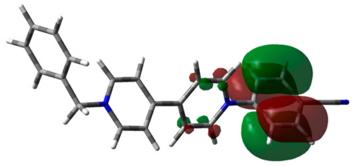	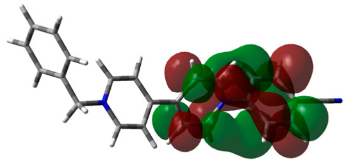
$W2a^{2+}$	269	S_1 (95%)	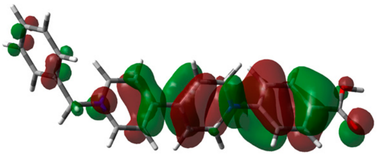	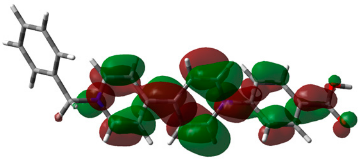
	234	S_7 (99%)	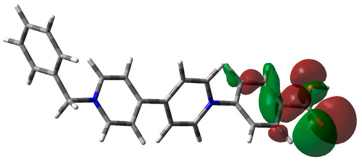	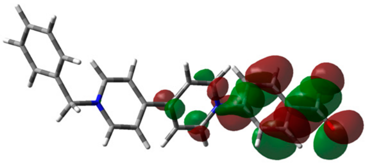
	199	S_{14} (72%)	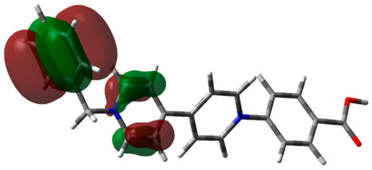	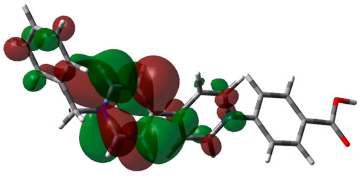
	196	S_{16} (84%)	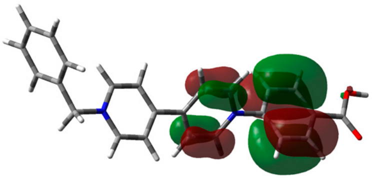	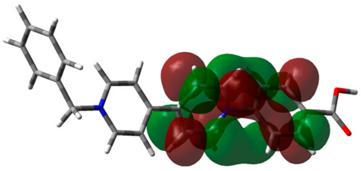
$W3a^{2+}$	268	S_1 (95%)	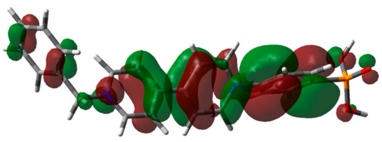	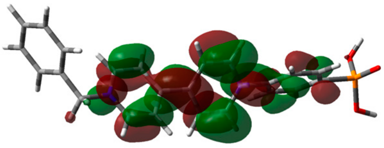

Continuation Table S15.

Cpds	λ, nm	State (% contribution)	HONTO	LUNTO
	231	S_7 (90%)	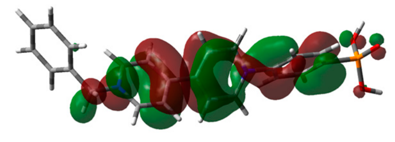	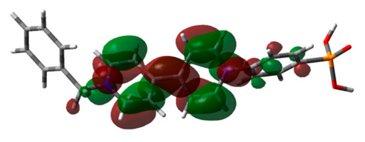
	199	S_{13} (72%)	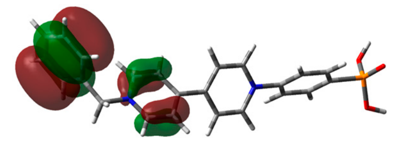	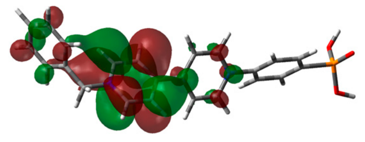
	194	S_{17} (65%)	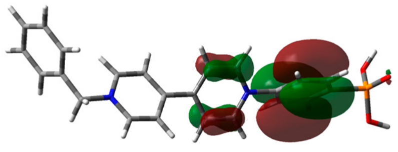	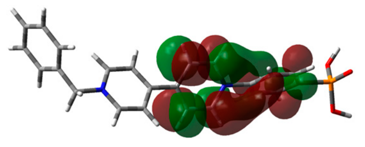
	188	S_{20} (62%)	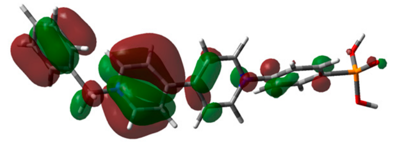	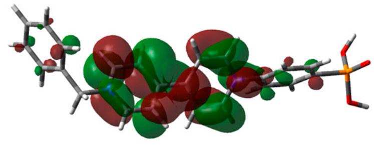
D1a^{2+}	299	S_1 (99%)	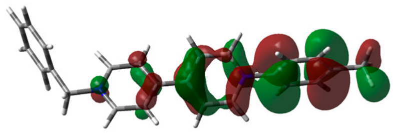	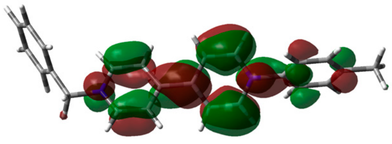
	237	S_7 (92%)	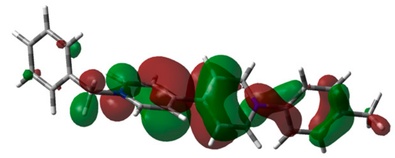	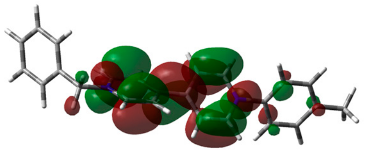
	199	S_{15} (72%)	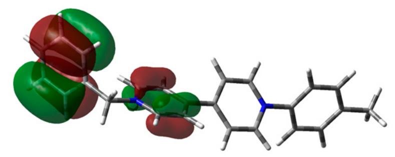	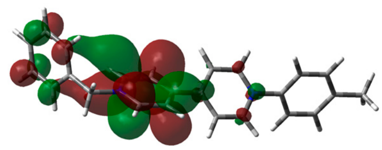
	192	S_{19} (57%)	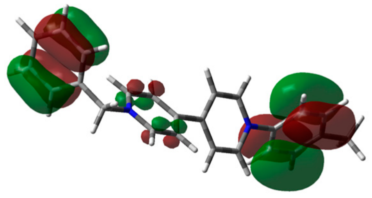	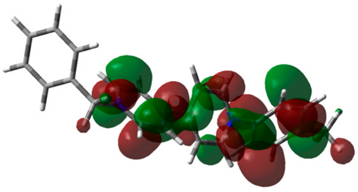

Continuation Table S15.

Cpds	λ, nm	State (% contribution)	HONTO	LUNTO
	328	S_1 (99%)	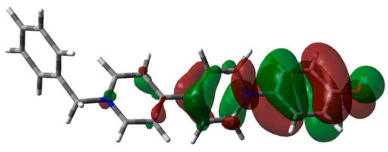	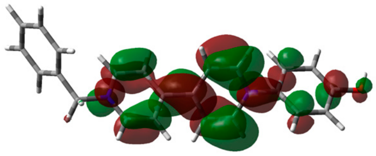
D2a^{2+}	237	S_8 (75%)	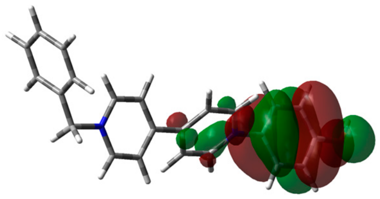	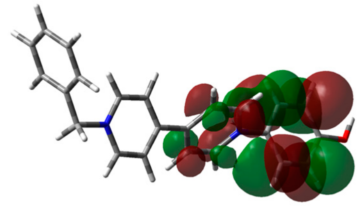
	199	S_{15} (72%)	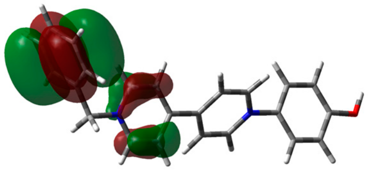	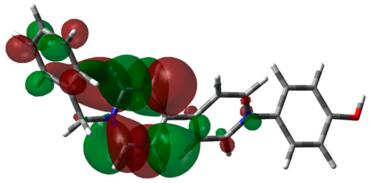
	405	S_1 (99%)	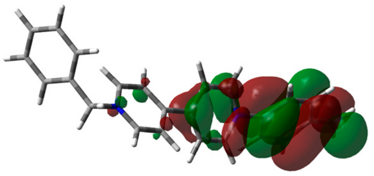	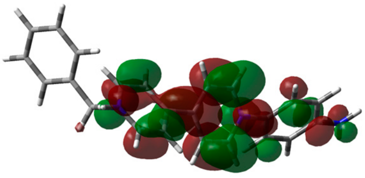
D3a^{2+}	240	S_{10} (92%)	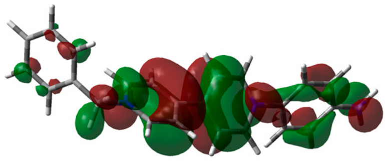	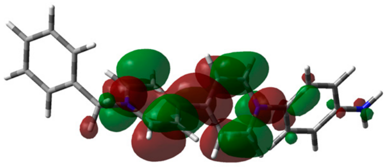
	198	S_{18} (73%)	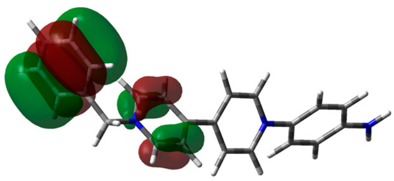	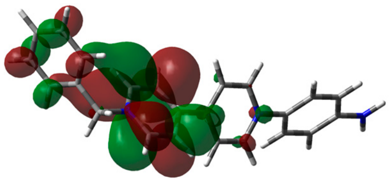

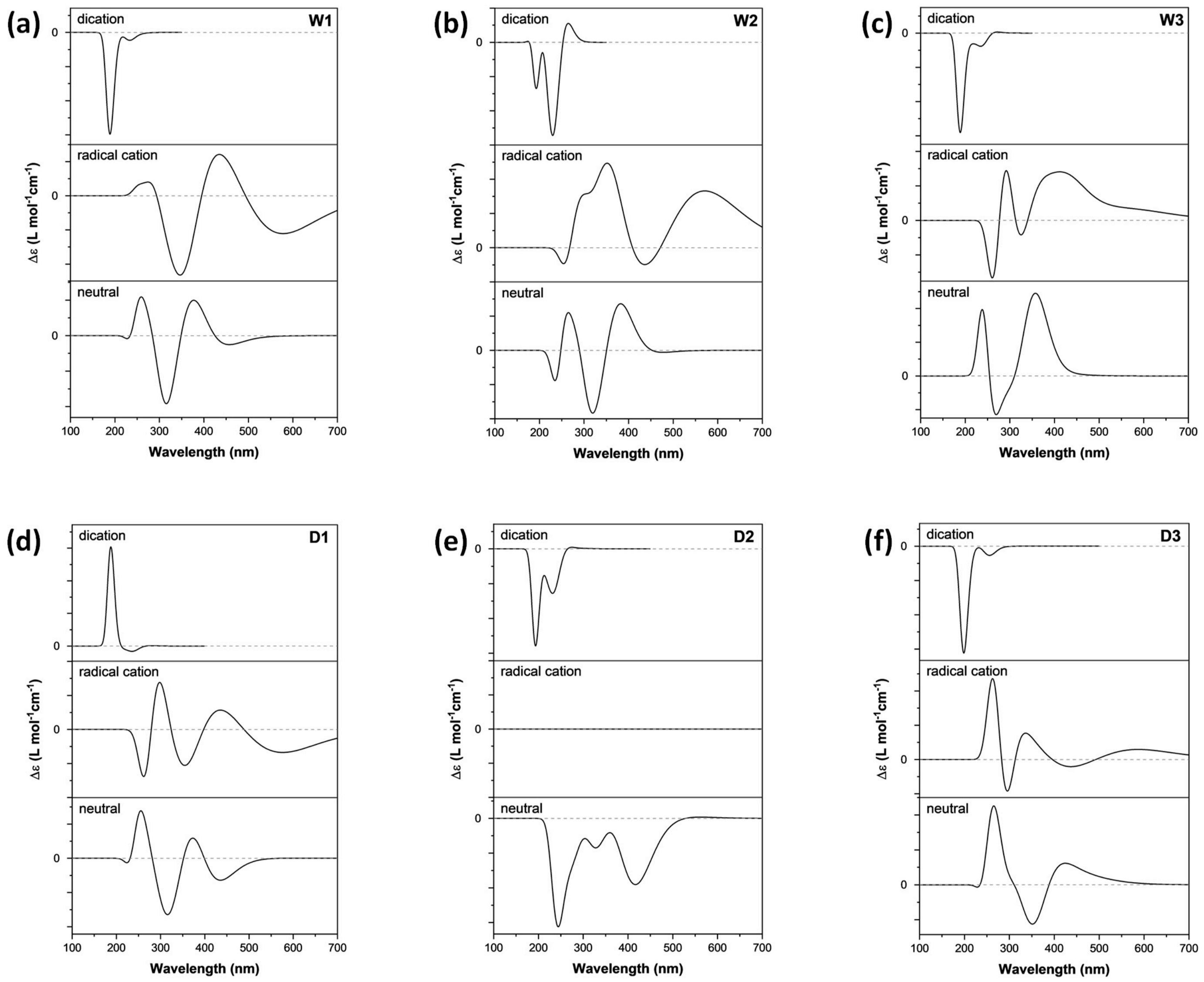

Figure S1. Simulated ECD absorption spectra of the designed Bn-V-Bn viologen derivatives: (a) W1 (Bn-V-Bn-CN); (b) W2 (Bn-V-Bn-COOH); (c) W3 (Bn-V-Bn-PO_3H_2); (d) D1 (Bn-V-Bn-CH_3); (e) D2 (Bn-V-Bn-OH); and (f) D3 (Bn-V-Bn-NH_2). The neutral structure of each derivative is shown as an inset.

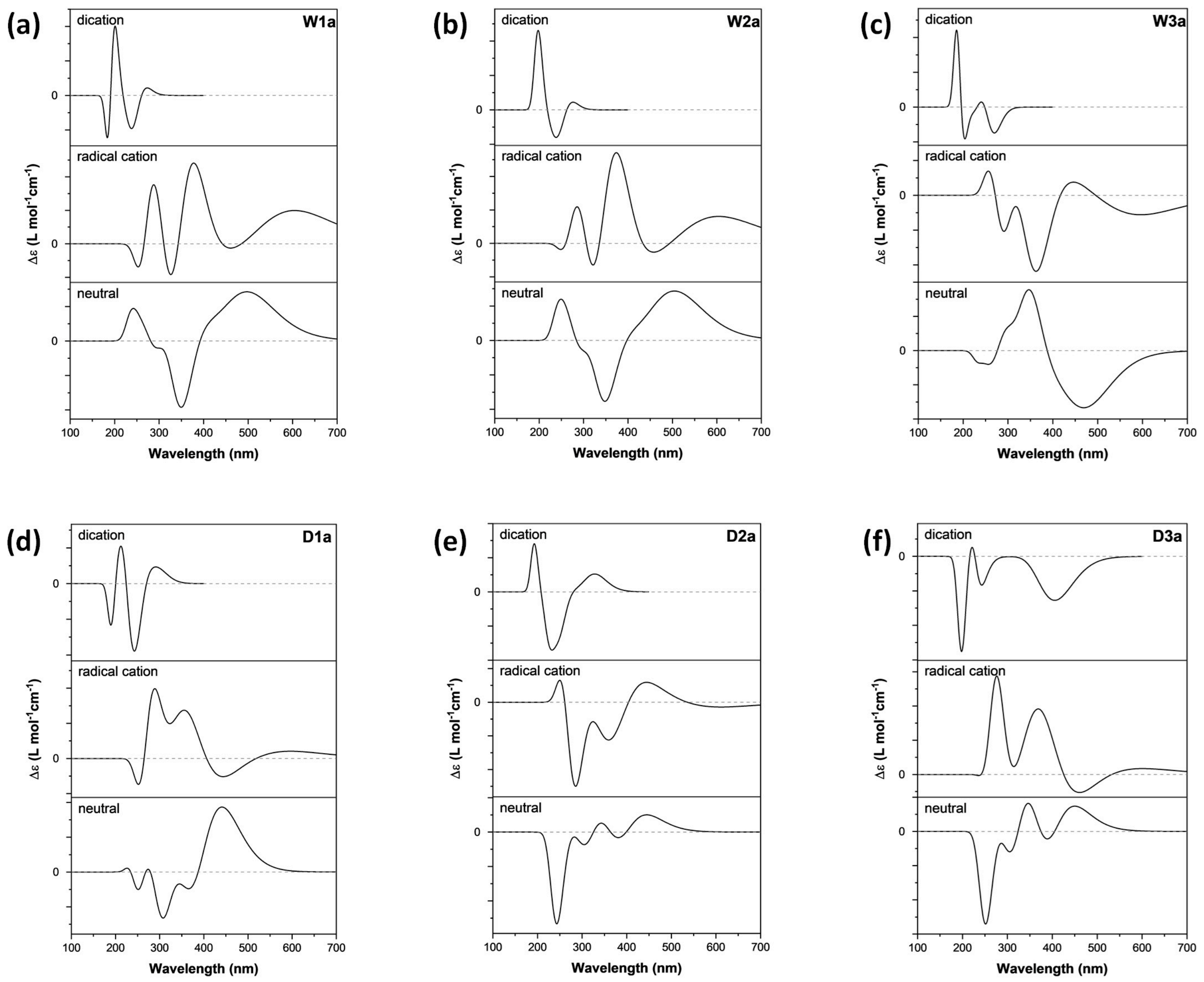

Figure S2. Simulated ECD absorption spectra of the designed Bn-V-Ph viologen derivatives: (a) W1a (Bn-V-Ph-CN); (b) W2a (Bn-V-Ph-COOH); (c) W3a (Bn-V-Ph-PO_3H_2); (d) D1a (Bn-V-Ph-CH_3); (e) D2a (Bn-V-Ph-OH); and (f) D3a (Bn-V-Ph-NH_2). The neutral structure of each derivative is shown as an inset.

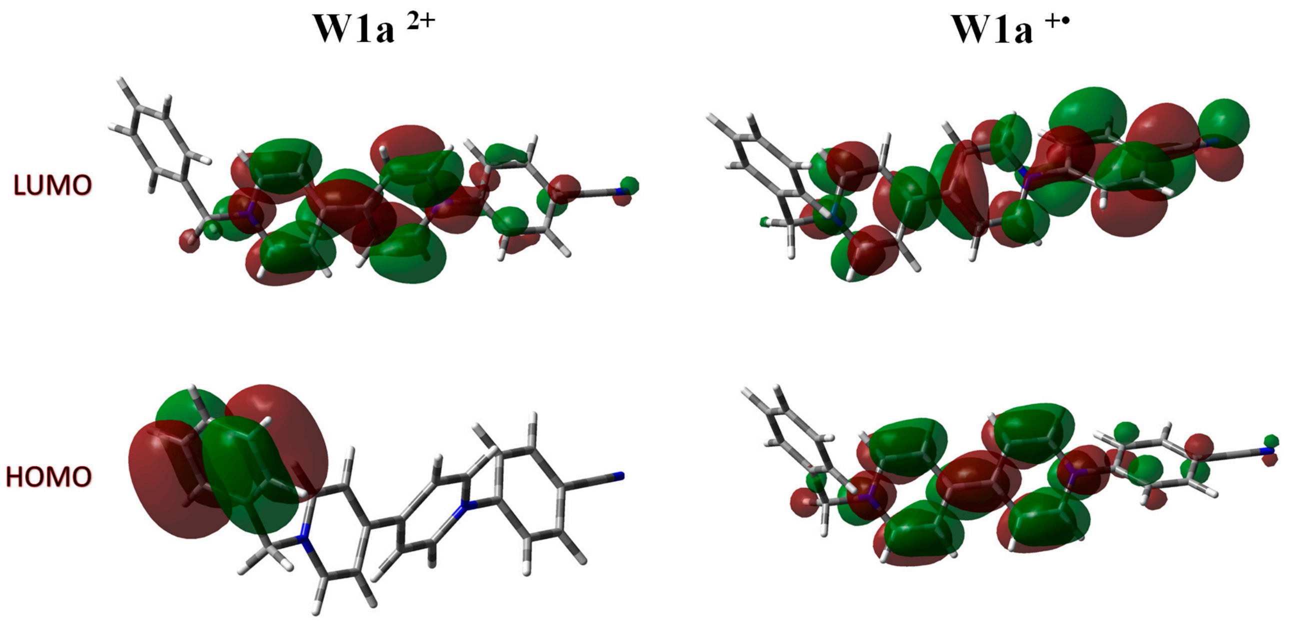

Figure S3. Visualization of HOMO and LUMO Orbitals of W1a (Bn-V-Ph-CN) and its oxidation states.

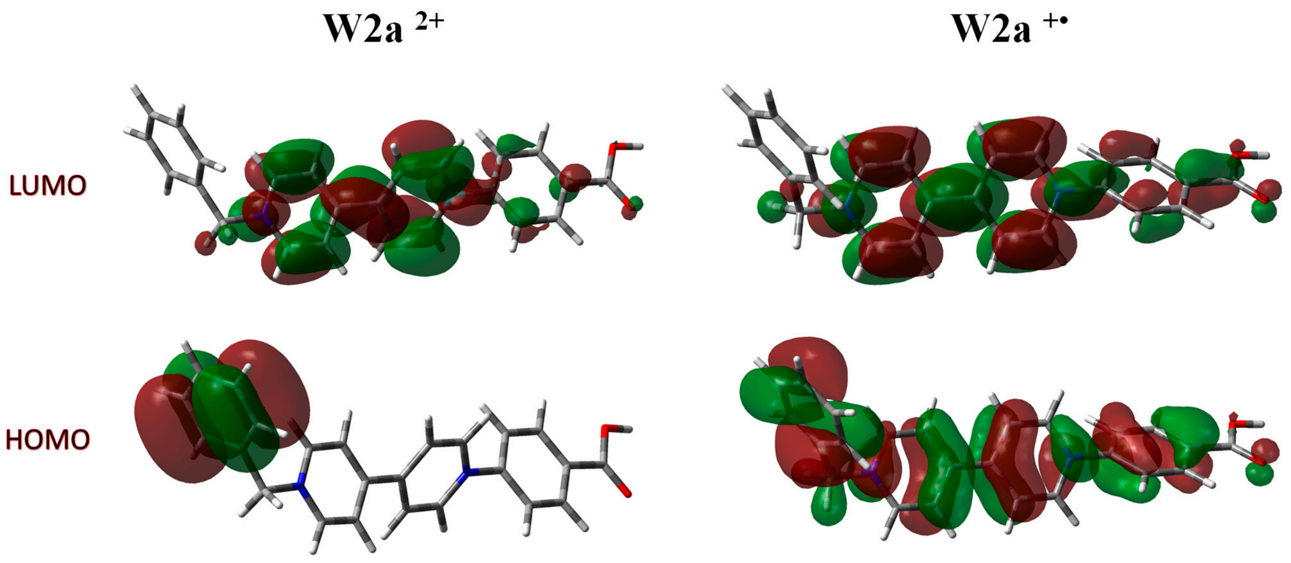

Figure S4. Visualization of HOMO and LUMO Orbitals of W2a (Bn-V-Ph-COOH) and its oxidation states.

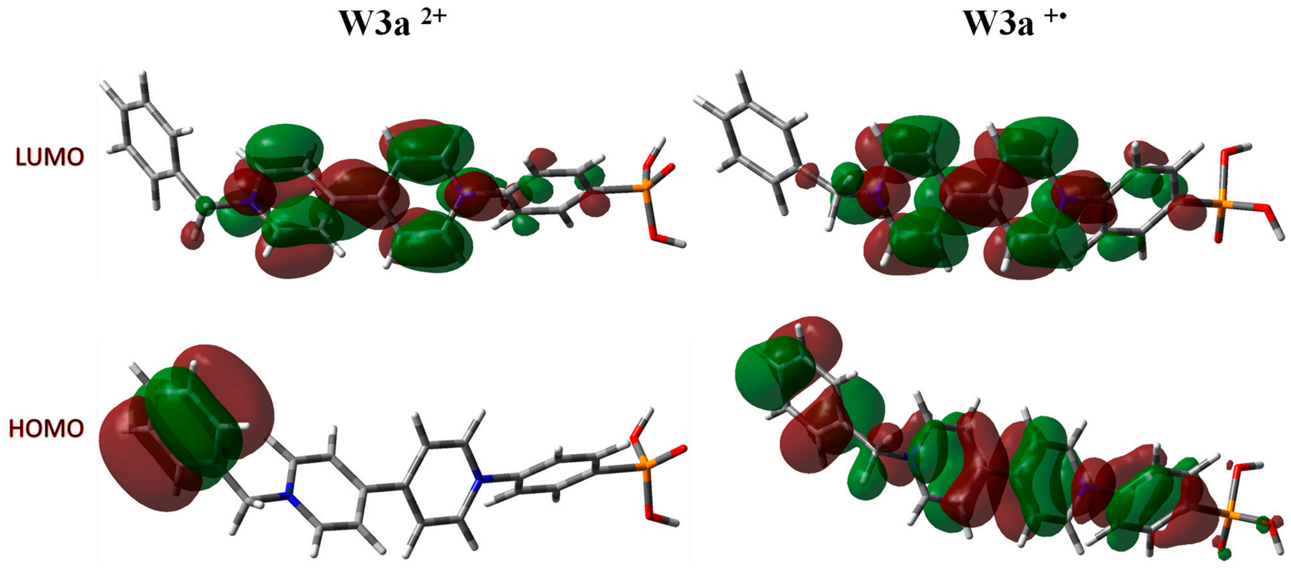

Figure S5. Visualization of HOMO and LUMO Orbitals of W3a (Bn-V-Ph-PO_3H_2) and its oxidation states.

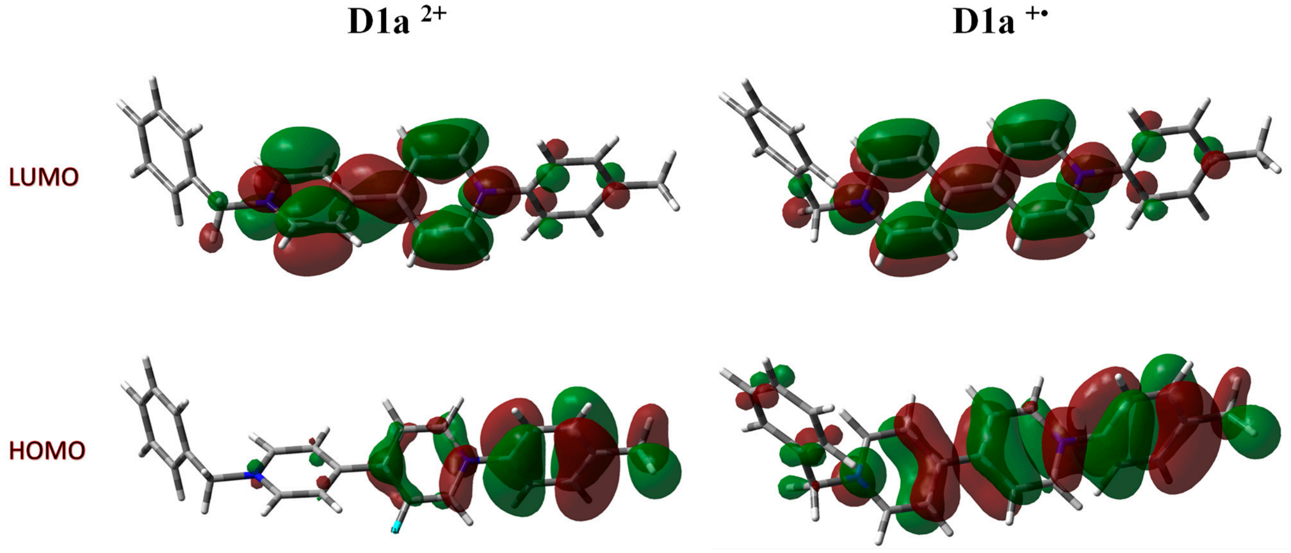

Figure S6. Visualization of HOMO and LUMO Orbitals of D1a (Bn-V-Ph-CH_3) and its oxidation states.

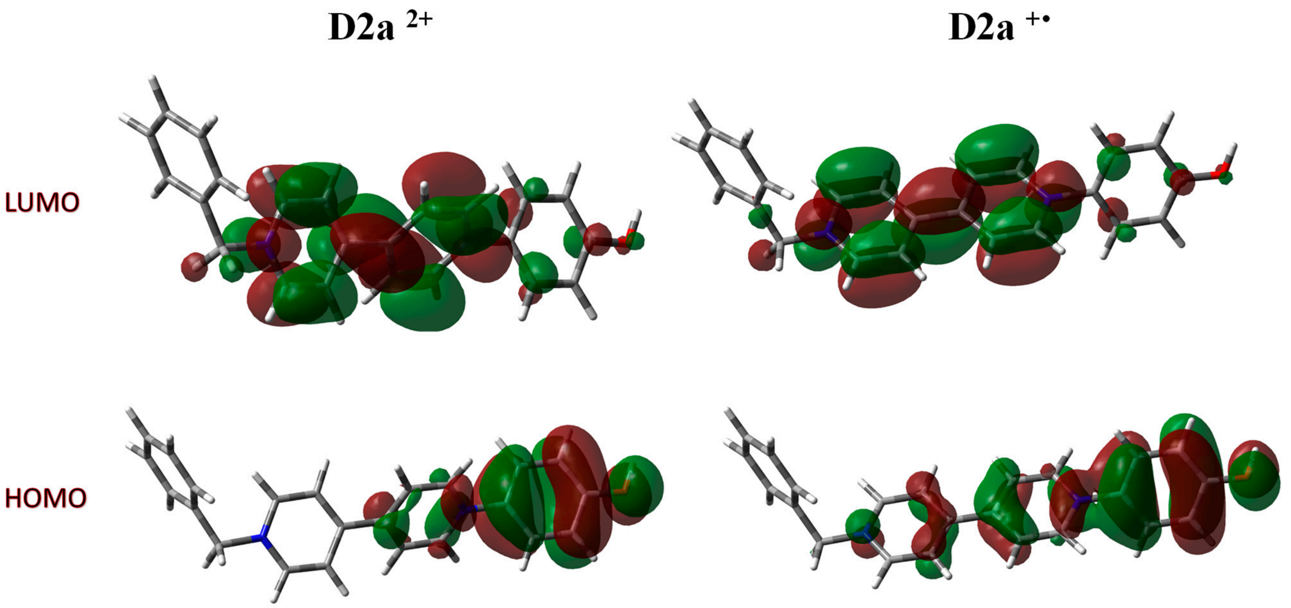

Figure S7. Visualization of HOMO and LUMO Orbitals of D2a (Bn-V-Ph-OH) and its oxidation states.

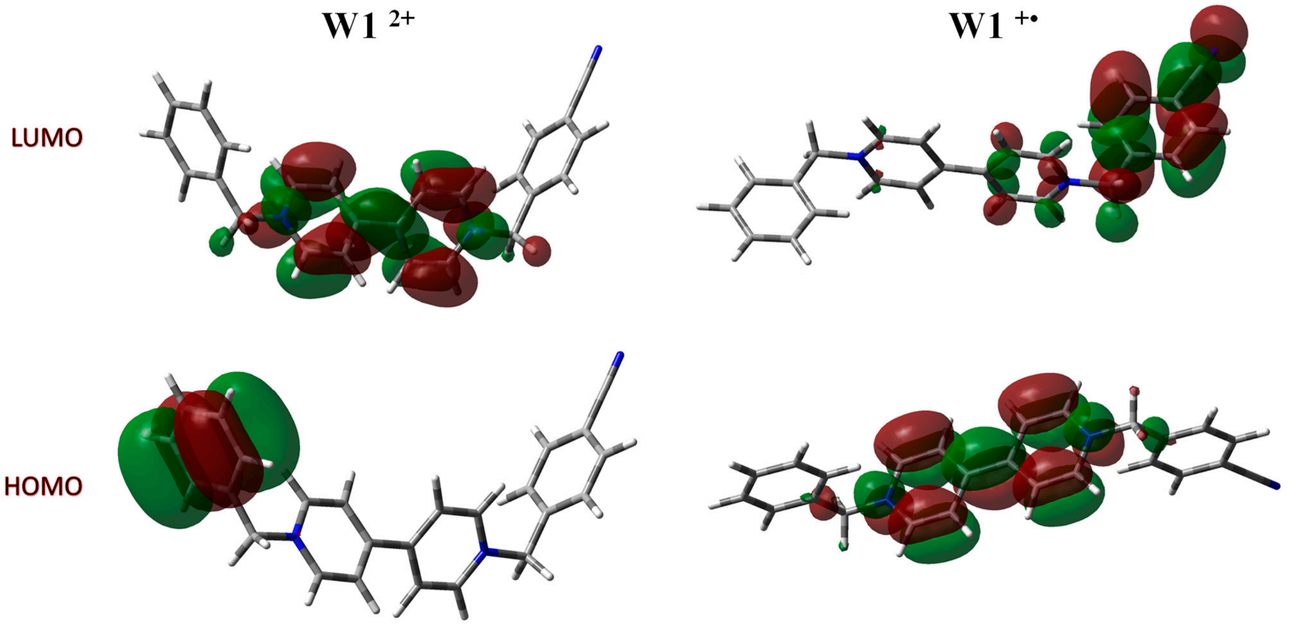

Figure S8. Visualization of HOMO and LUMO Orbitals of W1 (Bn-V-Bn-CN) and its oxidation states.

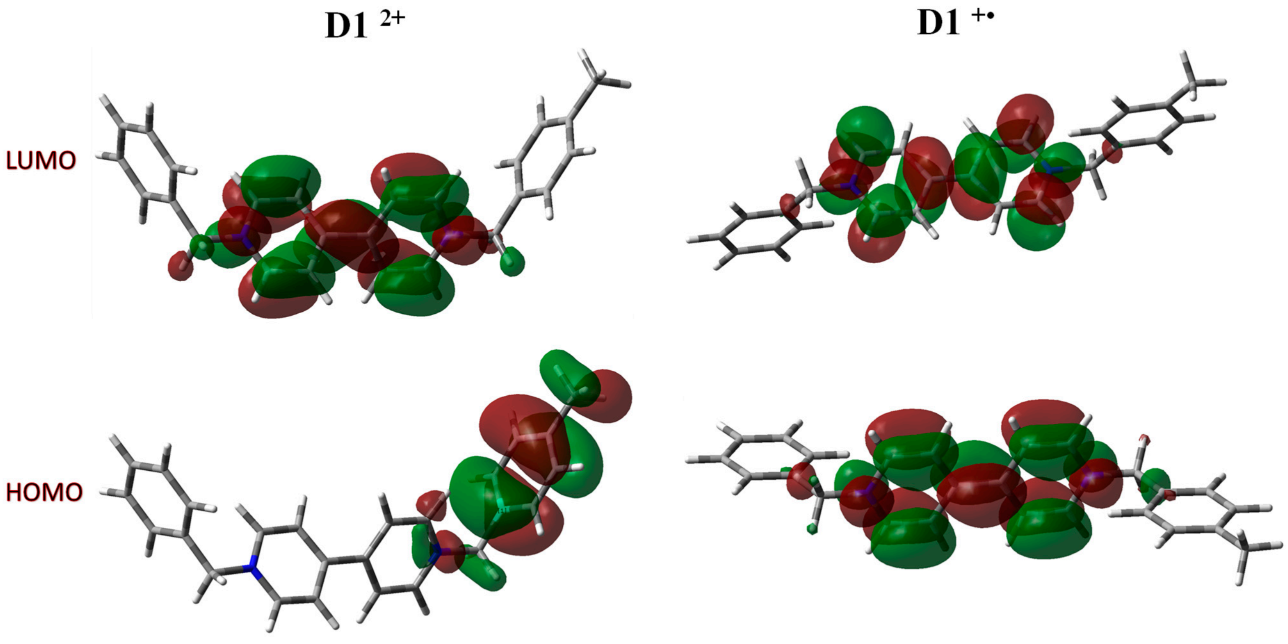

Figure S9. Visualization of HOMO and LUMO Orbitals of D1 (Bn-V-Bn-CH_3) and its oxidation states.

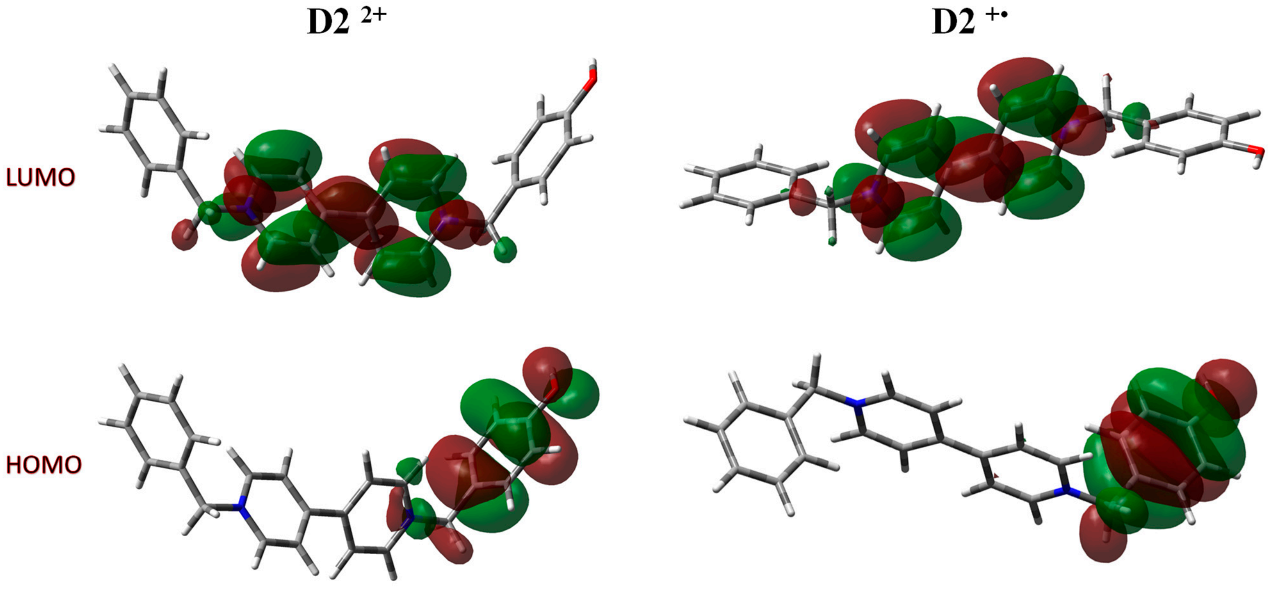

Figure S10. Visualization of HOMO and LUMO Orbitals of D2 (Bn-V-Bn-OH) and its oxidation states.

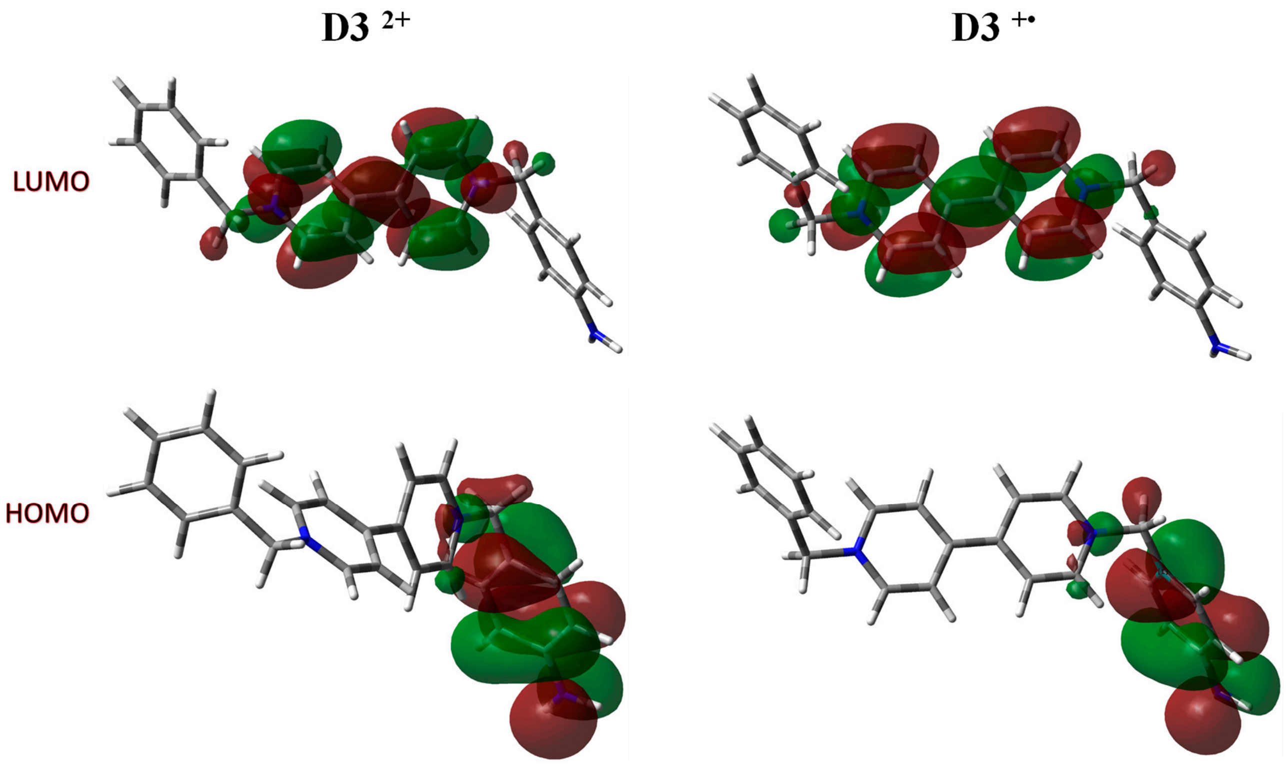

Figure S11. Visualization of HOMO and LUMO Orbitals of D3 (Bn-V-Bn-NH_2) and its oxidation states.
